# Anti-Obesity Drug Delivery Systems: Recent Progress and Challenges

**DOI:** 10.3390/pharmaceutics15112635

**Published:** 2023-11-16

**Authors:** Mohamed M. Ashour, Mostafa Mabrouk, Mohamed A. Aboelnasr, Hanan H. Beherei, Khairy M. Tohamy, Diganta B. Das

**Affiliations:** 1 School of Biotechnology, Badr University in Cairo, Badr City, Cairo 11829, Egypt; mohamedashour739@gmail.com; 2 Refractories, Ceramics and Building Materials Department, National Research Centre, 33 El Bohouth St., Dokki, Giza 12622, Egypt; hananh.beherei@gmail.com; 3 Biophysics Branch, Faculty of Science, Al-Azhar University, Nasr City, Cairo 11884, Egypt; abomalk3939@gmail.com (M.A.A.); already_a555@yahoo.com (K.M.T.); 4 Department of Chemical Engineering, Loughborough University, Loughborough LE113TU, UK

**Keywords:** anti-obesity drugs, active molecules, microneedles, nanoparticles, natural ingredients

## Abstract

Obesity has reached an epidemic proportion in the last thirty years, and it is recognized as a major health issue in modern society now with the possibility of serious social and economic consequences. By the year 2030, nearly 60% of the global population may be obese or overweight, which emphasizes a need for novel obesity treatments. Various traditional approaches, such as pharmacotherapy and bariatric surgery, have been utilized in clinical settings to treat obesity. However, these methods frequently show the possibility of side effects while remaining ineffective. There is, therefore, an urgent need for alternative obesity treatments with improved efficacy and specificity. Polymeric materials and chemical strategies are employed in emerging drug delivery systems (DDSs) to enhance therapy effectiveness and specificity by stabilizing and controlling the release of active molecules such as natural ingredients. Designing DDSs is currently a top priority research objective with an eye towards creating obesity treatment approaches. In reality, the most recent trends in the literature demonstrate that there are not enough in-depth reviews that emphasize the current knowledge based on the creation and design of DDSs for obesity treatment. It is also observed in the existing literature that a complex interplay of different physical and chemical parameters must be considered carefully to determine the effectiveness of the DDSs, including microneedles, for obesity treatment. Additionally, it is observed that these properties depend on how the DDS is synthesized. Although many studies are at the animal-study stage, the use of more advanced DDS techniques would significantly enhance the development of safe and efficient treatment approaches for obese people in the future. Considering these, this review provides an overview of the current anti-obesity treatment approaches as well as the conventional anti-obesity therapeutics. The article aims to conduct an in-depth discussion on the current trends in obesity treatment approaches. Filling in this knowledge gap will lead to a greater understanding of the safest ways to manage obesity.

## 1. Introduction

Obesity is a multifaceted issue that is of significant public health importance worldwide. It has the potential to impact individuals across all ages and socioeconomic strata and poses risks to all nations [1,2]. Obesity, and being overweight, refer to the buildup of an excessive amount of fat in an individual’s body with body mass indexes (BMI) of ≥30 and 25–29.9, respectively [2,3].

Back in 1995, the World Health Organization (WHO) estimated that around 200 million adults were obese, and 18 million children were overweight, around the world. Later in 2000, the number of obese adults escalated by over 300 million, according to the WHO’s 2005 report [1,2]. In 2016, there were more than 1.9 billion overweight adults, with over 650 million of them being labelled as obese. This means that about 13% of the global adult population was obese, with women having a higher obese percentage (15%) compared to men (11%). Furthermore, around 79% of the world’s adult population aged 18 and above were overweight during that year, with the men and women comprising of 39% and 40% of that population, respectively [4]. From 1975 to 2016, there was a significant increase in the worldwide occurrence of obesity, which nearly tripled.

**Figure 1:**
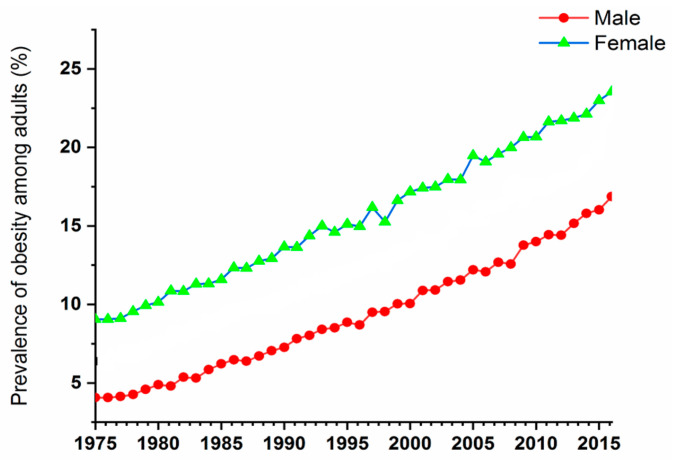
The frequency of obesity among individuals who are 18 years of age or older in the adult population from 1975 to 2016 according to the WHO [4].

In 2019, there was a total of 38.2 million children under the age of five who were identified as overweight or obese. While these issues were initially associated with high-income countries, they are now becoming more prevalent in metropolitan areas of low- and middle-income nations. For example, the proportion of under-fives who are overweight is increasing (WHO 2022) in Africa [4]. The prevalence of obesity among adults in the time period of 1975–2015 is demonstrated in Figure 1. It is clear that women are showing higher numbers during this period; however, the numbers of both males and females are significantly increasing with time. These numbers have encouraged scientists to start looking for treatments for people suffering from obesity and its complications.

**Figure 2:**
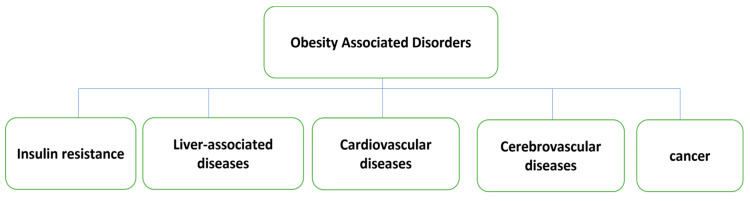
Chart of obesity-associated diseases.

Obesity is strongly related to a variety of life-threatening conditions such as diabetes, heart disease, sleep apnoea, various types of cancers, osteoarthritis, etc. [5,6,7,8]. It causes many other diseases that include central nervous system (CNS) diseases such as Alzheimer’s disease and depression [9,10,11], respiratory problems such as emphysema and chronic bronchitis [12,13], cardiovascular diseases such as atherosclerosis and hypertension [14], digestive diseases such as fatty liver and ulcerative colitis, bone and joint diseases such as osteoarthritis [15,16], and metabolic diseases such as diabetes and gout [17,18]. Obesity can also cause male sexual dysfunction, kidney disease, irregular menstruation, and female infertility [19,20]. Obesity-associated diseases are demonstrated in Figure 2.

Obesity is well known to cause a variety of cancers such as endometrial cancer, colorectal cancer, stomach cancer, breast cancer, liver cancer, and others [21,22,23]. Obese patients have a higher risk of inflammation, lower immunity, and premature aging [24]. Obese people with a BMI greater than 27 kg/m^2^ and other obesity-related complications must be treated with medication [24]. In the case of obese individuals, excessive secretion of pro-inflammatory adipokines by adipocytes within adipose tissue can lead to a systemic inflammatory state. Additionally, the hydrolysis of triglycerides in adipose cells releases free fatty acids that are transported to where they can be utilized metabolically. However, in obese patients, there are elevated levels of fatty acids and cholesterol, resulting in a greater mass of adipose cells, causing an increase in their size and volume. Although the lipids can be found in adipose tissue, they are also present in a variety of cell types in the form of small cytoplasmic organelles called liposomes, which can lead to the expansion of liver tissue and cause various pathological conditions like non-alcoholic fatty liver disease, steatohepatitis, and cirrhosis. In some non-fatty tissues, excessive amounts of lipoidal intermediates can cause cell depletion and death through lipotoxicity. In overweight or obese patients, elevated levels of free fatty acids, inflammatory proteins, and lipoidal intermediates in non-adipose tissues can compromise insulin resistance and signalling. Additionally, there is a strong link between excess intra-abdominal fat and insulin resistance. A high number of white adipocytes, which retain various triglycerides, are present in subcutaneous fat cells, while a relatively small and consistent number of brown and beige adipocytes with thermogenic properties exist in adults. When adipose tissue undergoes modifications due to adipocyte cell death, obesity is often accompanied by an increase in immune cells, particularly macrophages. These immune cells release pro-inflammatory proteins as cell signalling molecules, which contribute to the insulin resistance commonly observed in obese individuals [25].

**Figure 3:**
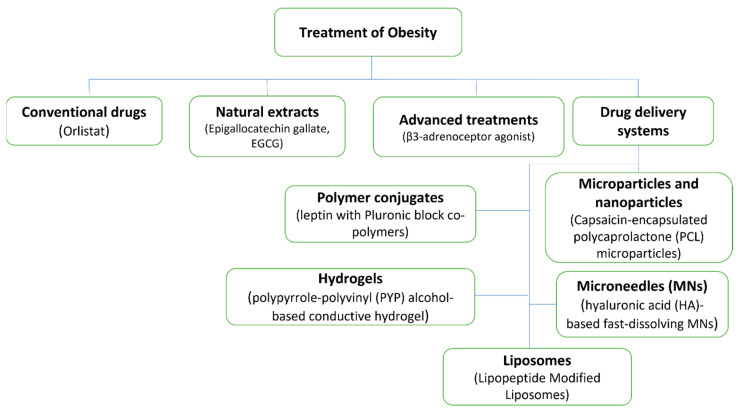
Obesity treatment approaches chart.

Obesity is a treatable disease that can be managed by either physical or therapeutic control, as discussed briefly below. Different obesity treatments are illustrated in Figure 3, including classical treatment techniques using natural products and advanced techniques that include different DDSs.

Physical control: Obesity is primarily managed through dieting and physical activity [2]. Obese and overweight people must follow a strict diet plan and engage in strenuous exercise. Regular diet program maintenance is typically challenging, and in a majority of cases, a person must adhere to these lifestyles indefinitely [26,27]. A low-calorie diet and strenuous exercise have a variety of negative effects, including an increased risk of loss of lean muscle mass and gout. A person who follows this lifestyle for a long time should be monitored by a physician to avoid complications [28]. However, only 2–20% of long-term weight loss attempts involving lifestyle changes are successful [29].

The non-therapeutic treatments are essential for the management of obesity, as they can help individuals achieve and maintain healthy weights. Non-pharmacological treatments for obesity include behavioural and lifestyle modifications, such as increased physical activity, dietary changes, and behavioural therapy. These interventions have been shown to be effective in reducing body weight and improving overall health. A healthy diet for weight loss typically involves reducing calorie intake and increasing the consumption of nutrient-dense foods. Regular physical activity helps to burn calories, increase muscle mass, and improve overall health. Behavioural therapy helps individuals to identify and modify unhealthy behaviours that contribute to weight gain, such as overeating and a sedentary lifestyle [30].

Therapeutic control: The most effective obesity treatment is bariatric surgery. Due to several complications after surgery, only those who are extremely obese should consider this surgery (BMI > 40) [31]. There are only a limited number of therapeutic substances, like Orlistat, Sibutramine, Lorcaserin and Phentermine/Topiramate, that have the ability to decrease body weight by either reducing food intake and absorption or increasing energy expenditure [32,33]. Regrettably, the effectiveness of these medications in regulating body weight has been restricted, and the majority of them have been removed from the market due to severe adverse reactions [34]. Comprehensive research is required to gain a better understanding of the development of obesity and to identify the safe and effective therapeutic methods for controlling it, due to the current state of obesity, its associated conditions, as well as the limitations of obesity drugs [34].

Paul Ehrlich developed the idea of targeted drug delivery systems as “magic bullets” nearly a century ago, which deliver medication to their target organ while preventing it from affecting healthy organs of the body [35]. Targeted drug delivery is also known as smart drug delivery [36]. An intensive effort has been directed over the last three decades towards the advancement of drug delivery systems (DDSs) for disease treatment. A DDS can be characterized as a technique for effectively delivering the medication to its therapeutic site of action by choosing the right carrier, route, and target. The selection of these three critical factors determines the efficacy of the DDS.

Employing a carrier system to transport medication within the body presents various possibilities for successfully achieving the objective of drug targeting. Some of the potential benefits of DDSs are as follows [35,36,37]:

- Maintaining constant drug levels within the therapeutic range.
- When drugs are targeted to specific tissues/organs, they have less toxicity and fewer side effects.
- Administration is made easier, which increases patient compliance.
- Defence against the degradation of biologically active drug particles such as proteins and peptides.
- Small doses of the drug and a reduction in the number of dosages.

Keeping the above trends in consideration, this review paper aims to clarify various methods for treating obesity by discussing traditional medicines as well as the treatment methods that have been used most recently to treat this common disease. Possible applications of DDSs such as nanoparticles (NPs) and microneedles (MNs) have been discussed to fill the gap between traditional and recent treatment approaches and provide more in-depth knowledge of how obesity can be managed safely. The review structure includes an introduction to the disease of obesity and the increasing number of people infected with it based on the data provided by the World Health Organization, as well as the symptoms related to this disease, methods, treatments, traditional methods, and health problems associated with these medicines and their danger to the public health of users of these medicines. These factors led to the necessity of searching for methods and treatments that are safer for the health of patients, until the drug delivery techniques were developed, which have proven their effectiveness in treating obesity as well as their safety. The literature for this review has been selected from the literature search engine Scopus, and the main keywords used to select the papers were “obesity treatment”, “microneedles”, “obesity associated disorders”, “conventional obesity drugs”, “natural ingredients”, “advanced treatment” and “drug delivery systems”.

## 2. Conventional Anti-Obesity Drugs

Anti-obesity drugs are still primarily administered orally or via injection. The US Food and Drug Administration (FDA) has recommended five types of anti-obesity drugs: Orlistat, Phentermine/Topiramate ER, Naltrexone SR/Bupropion SR, Lorcaserin, and Liraglutide. By using the current delivery method, the efficacy of these drugs has only been found to be 3–7%. At the moment, Liraglutide is administered intravenously, whereas all other drugs are administered orally [38,39]. Common anti-obesity drugs alongside their principles of action, delivery mode, and side effects are listed in Table 1.

The major effects of Phentermine/Topiramate ER, Naltrexone SR/Bupropion SR, Lorcaserin, and Liraglutide are to reduce calorie intake by controlling appetite and boosting satiety. Furthermore, investigation on the precise mechanism is ongoing [40]. These medications have a variety of side effects, including high blood pressure, arrhythmia, nausea, dizziness, insomnia, taste failure, constipation, and so on. As a result, using these medications is frequently disallowed in patients with cardiovascular diseases, people taking other medications, and pregnant women [41]. Orlistat is a lipase inhibitor, which checks fat absorption in the body, reduces calorie intake, and regulates weight gain [38]. Orlistat is the world’s bestselling over-the-counter (OTC) anti-obesity drug, and it is safe for teenagers [42].

**Table 1:** Known principles, delivery modes, and side effects of common anti-obesity drugs.

Action Principle	Delivery Method	Name of Drug	Drawbacks	Reference
Reduce appetite	Oral administration	Phentermine/Topiramate ER	Taste disorders, insomnia, dizziness and constipation	[43]
		Naltrexone SR/Bupropion SR	Headache, diarrhoea and constipation	[43]
		Lorcaserin	Nausea, dizziness and constipation	[44]
		Rimonabant	Discomfort, nausea and gastrointestinal distress	[43]
		Fenfluramine	Hypertension and heart valve damage	[43]
	Hypodermic injection	Liraglutide	Neuropsychiatric diseases, depression and dizziness	[38,39]
Block the absorption of fat	Oral administration	Orlistat	Flatulence and diarrhoea	[42]
Increase energy consumption and reduce appetite		Sibutramine	Cerebrovascular diseases and cardiovascular	[43]

Although these drugs are widely used for losing weight, they do have some drawbacks, including diarrhoea and flatulence. It has also been demonstrated that metformin aids in weight loss. For patients with type 2 diabetes who were obese, metformin was the first oral medication of choice. Constipation and stomach aches are among its reported side effects [45,46,47]. There are numerous drawbacks to these drugs entering the human body via injection or the traditional oral route, such as limited effectiveness, some side effects, large doses, patient non-compliance, and inconvenience of use. Some FAD-approved oral anti-obesity medications are no longer available. The FDA released a caution in early 2020 that the weight loss medications BelviqXR (Lorcaserin) and Belviq could increase the risk of cancer in obese patients, but it is unclear whether the drugs will be prohibited [44]. The administration of drugs throughout the body is believed to be a significant factor in producing a range of undesirable effects that arise due to excessive dosages. These effects can significantly impede the availability of the medication [48].

## 3. Advanced Treatments of Obesity

An imbalance between caloric intake and consumption is what leads to obesity. Adipocytes will store excess caloric intake that cannot be consumed promptly [49]. In addition to suppressing appetite, increasing the feeling of fullness, and preventing the absorption of nutrients, the latest medications for treating obesity also target the promotion of heat production or breakdown of fat in adipocytes [50,51]. Most adipose tissue (AT) is found in the subcutaneous and visceral organs. With over 80% of total body fat stored in the subcutaneous tissue, treating obese patients by lowering localized subcutaneous AT is very useful [52]. White adipose tissue (WAT) and brown adipose tissue (BAT) are the two types of adipose tissue [53]. The process of “browning” causes white fat cells to transform into brown-like adipocytes through various drug-related triggers [54].

The new anti-obesity agents and their delivery methods alongside their mechanism of actions are illustrated in Table 2.

WAT’s nature has been considered one of the reasons why there are so many difficulties with obesity; WAT’s main function is to store energy, of which there is typically more than there should in obese patients [55]. In contrast to WAT, brown fat cells found in BAT are metabolically active, which produces heat and raises energy consumption in the body. The primary protein responsible for this process as a thermogenic protein is known as uncoupling protein 1 (UCP1). One potential method for combating obesity is targeting WAT and converting it into cells resembling brown adipocytes. This approach is believed to have great potential for increasing energy expenditure in humans [56,57,58].

Several substances, including β3-adrenoceptor agonists (CL316243), thyroid hormone (T3), rosiglitazone (ROSI), bile acid, fucoxanthin, curcumin, and others, have been shown to enhance browning and, thus, to increase thermogenesis [49,59,60,61]. Gelatine, gold NPs, and caffeine have all been shown to aid in fat decomposition [62,63,64]. It was discovered that glucagon-like peptide 1 (GLP1) analogues and resveratrol may activate brown adipocytes to promote not only thermogenesis, but also browning [48,65,66]. Some researchers began to investigate anti-obesity medications delivered transdermally to enhance their effectiveness and prevent adverse effects brought on by DDSs.

CL316243 was found to enhance adipocyte browning in obese mice [67]. Mirabegron (β3-adrenoceptor agonist) has been licensed by the FDA for overactive bladder treatment. Frequent usage of the medication, in addition, can cause an increase in blood pressure and heart rate [68]. The thyroid hormone T3 has been shown to induce fat browning and thermogenesis. It has been observed that T3 or T4 causes loss of weight in humans and animals [69,70]. Long-term frequent usage of thyroid hormone could cause cardiovascular disease and hyperthyroidism [71], which is why it has not been licensed as an anti-obesity medicine.

The peroxisome proliferator-activated receptor (PPAR), which is responsible for regulating the storage of fatty acids and glucose metabolism, is also a crucial transcriptional regulator for the synthesis of BAT [72]. ROSI is a type of PPAR activator that can increase insulin sensitivity in AT, liver, and skeletal muscle, and it has been utilized for diabetes treatment [73]. Recent research has revealed that it also has a browning effect [60]. However, taking ROSI may increase the risk of cardiovascular disease [74].

**Table 2:** Possible principles of actions and delivery methods of new anti-obesity agents.

Action Principle	Delivery Mode	Name of Drug	References
Activation of brown adipocytes	Transdermal and hypodermic injection	β3-adrenoceptor agonist (CL316243)	[67,75]
		thyroid hormone (T3)	[76,77]
	Transdermal and oral administration	ROSI	[73,78]
		curcumin	[79,80]
	Oral administration	fucoxanthin	[81]
		bile acid	[82]
		capsaicin	[83]
		olive oil	[84]
	Hypodermic injection	GLP1 analogue	[65]
	Oral administration	resveratrol	[48]
Fat decomposition	Transdermal and oral administration	gelatine	[62]
		caffeine	[64,85]
	Transdermal administration	gold NPs	[63,86]

## 4. Natural Anti-Obesity Extracts

Natural products, such as animal-derived natural products (e.g., fish oil and chitosan from crab and shrimp shells) and plant-derived natural products (e.g., citrus limon and Panax ginseng) have been reported to reduce obesity-related metabolic disorders [87,88,89,90,91,92,93,94,95,96,97,98,99,100,101,102,103,104,105,106,107,108,109,110,111,112,113,114,115,116,117,118,119,120,121,122,123,124,125,126,127,128,129,130,131,132,133,134,135,136,137,138,139,140,141,142,143,144]. A well-known therapeutic ingredient is green tea that is abundant in catechins, a type of polyphenol [92,93]. The primary catechin in green tea, (-)-epigallocatechin-3-gallate (EGCG), is thought to be a major factor in the health benefits of green tea, such as its ability to prevent cancer [94,95,96] and antimetabolic syndrome [97,98,99]. It also has antiviral and anti-infectious effects [100,101], protects the heart from cardiovascular diseases [102], and has neuroprotective effects [103]. In zebrafish models of diet-induced obesity, green tea extract (GTE) was found to lower total cholesterol (TCHO) levels and plasma triglyceride (TG) and visceral adipose tissue (VAT) volume in 2019 [104]. It was reported that people who consume green tea habitually were shown to have lower fat levels in numerous epidemiological analyses [98,105,106,107]. Natural anti-obesity agents and their active ingredients and mechanisms of action are demonstrated in Table 3.

It was found that natural extractions such as phenolic acids, flavans-3-ol (catechin), anthocyanins, curcuminoids, lignans, flavonols, iso-flavonoids, flavones, alkaloids (caffeine), and phytosterols have anti-obesity effects [108].

**Table 3:** Natural anti-obesity extractions.

Principle of Action	Natural Agent	Active Constituent	Reference
Pancreatic lipase inhibitor	Panax japonicus	Chikusetsusaponins	[109]
	Thea sinensis (oolong tea)	Crude aqueous extract (caffeine)	[110]
	Cassia mimosoides	Proanthocyanidin	[111]
	Trigonella foenum graecum L. (seed)	Crude ethanolic extract	[112]
	Salix matsudana (leaf)	Polyphenol	[113]
	Vitis vinifera	Crude ethanolic extract	[114]
	Salvia officinalis L. (leaf)	Methanolic extract (carnosic acid)	[115]
	Cassia nomame	Flavan dimers	[116]
	Citrus unshiu	Hesperidin	[117]
	Chitosan-chitin	Chitosan (80%), chitin (20%)	[118]
	Streptomyces toxytricini (fungus)	Lipistatin	[119]
	Actinomycetes sp.	Valilactone	[120]
	Caulerpa taxifolia (marine algae)	Caulerpenyne	[121]
Appetite suppressant	Panax ginseng (root)	Crude saponins	[122]
	Camellia sinensis (leaf)	(-)-Epigallocatechin gallate (EGCG)	[123]
	Hoodia gordonii and H. pilifera	Steroidal glycoside	[124]
	Haseolus vulgaris and Robiniapseudoacacia	Lectins	[125]
	Pinus koraiensis (pine nut)	Pine nut fatty acids	[126]
	Ephedra species	Ephedrine	[127]
	Citrus aurantium	Synephrine	[128]
	Hypericum perforatum	Total extract	[129]
Adipocyte differentiation inhibitor	Chili pepper (capsicum)	Capsaicin	[130]
	Fish oil	Docosahexaenoic acid	[131]
	Palm oil	Ɣ-tocotrienol	[132]
	Camellia sinensis (green tea)	(-)-Epigallocatechin gallate	[133]
	Panax ginseng	Ginsenosides	[134]
	Silybum marianum	Silibinin	[135]
	Garlic	Ajoene	[136]
	Rosmarinus officinalis	Carnosic acid	[137]
	Curcuma longa	Curcumin	[138]
	Humulus lupulus	Xanthohumol	[139]
Lipid metabolism regulator	Morus albam, Melissa officinalis, Artemisia capillaries	Crude aqueous extract	[140]
	Curcuma longa L.	Curcumin and curcuminoids	[141]
	Glycyrrhiza glabra L.	Liquorice flavonoid	[142]
	Panax ginseng	Crude aqueous extract	[143]
	Zea mays L.	Purple corn colour (anthocyanins)	[144]
	Soybean	Genistein and L-carnitine (soy isoflavone)	[145]
Energy expenditure stimulant	Solanum tuberosum	ethanolic extract	[146]
Lipid metabolism regulator and pancreatic lipase inhibitor	Coffea canephora	Caffeine and chlorogenic, neochlorogenic, and feruloyquinic acids	[147,148]
Appetite suppressant and adipocyte differentiation inhibitor	Garcinia cambogia	(-)-Hydroxycitric acid (HCA)	[149,150]

## 5. Advanced DDSs for the Treatment of Obesity

Because of their high bioavailability, low dose and side effects, and ease of administration, DDSs are ideal for the delivery of anti-obesity therapeutics [43]. The high targeting ability of DDSs appears to offer a high possibility for reducing local subcutaneous AT [151]. Despite significant efforts in recent years, conventional obesity treatment methods are frequently inadequate for maintaining metabolic balance and preventing potentially fatal consequences. New techniques for improving their effectiveness and reducing side effects are, thus, critical for obesity management. Advancements in biomaterials for the delivery of drugs are allowing substantial progress in therapy, with a variety of polymeric carriers that release medicines for prolonged periods, as well as further customized targeting of specific locations or cell types inside the body [152]. Polymer conjugates [153], hydrogels [154], MNs [78], micro- and NPs [155,156], and liposomes [157] represent a few polymeric carriers.

### 5.1. Preparation and Characterization of Anti-Obesity DDSs

In order to enhance the specificity of DDSs, different strategies can be employed. One of these methods involves incorporating molecular recognition elements as targeting entities. These targeting molecules can attach to receptors or biomarkers that are either overrepresented or particular to the target cells or tissues, which facilitates drug delivery to the desired site [158].

Antibodies are frequently utilized as targeting molecules as they can be modified or chosen to selectively attach to antigens located on the surface of target cells. By conjugating drugs or drug-loaded nanoparticles to these antibodies, the DDS can deliver the therapeutic agents to the desired cells, while avoiding healthy cells. This method is commonly known as antibody–drug conjugate (ADC) therapy [159].

Another targeting molecule that can be used is the peptide. Peptides are short chains of amino acids that can be designed to recognize and bind to specific receptors or biomarkers. These peptides can be either natural or synthetic and can be conjugated to drug molecules or used as carriers for drug-loaded nanoparticles. Peptide-based targeting has been explored in various disease conditions, including cancer, cardiovascular diseases, and neurological disorders [160].

In addition to antibodies and peptides, other targeting molecules such as aptamers, small molecules, and carbohydrates have also been investigated. Aptamers are short, single-stranded DNA or RNA molecules that can be selected to bind to specific targets with high affinity and specificity. Small molecules, on the other hand, are low-molecular-weight compounds that can be designed to bind to specific receptors or enzymes involved in disease pathways. Carbohydrates, especially glycan-based targeting, have gained attention due to their ability to recognize specific lectins or receptors on cell surfaces [161].

Furthermore, advancements in nanotechnology have led to the development of targeted DDSs using functionalized nanoparticles. These nanoparticles can be engineered to carry both targeting molecules and therapeutic agents, enabling specific delivery to the target cells or tissues. Surface modifications with targeting ligands, such as antibodies or peptides, allow for enhanced cellular uptake and specific accumulation at the target site.

The use of molecular recognition elements as targeting molecules in DDSs has the potential to improve the specificity and efficacy of drug delivery. By selectively delivering therapeutic agents to the desired cells or tissues, these targeted DDSs can enhance treatment effectiveness while minimizing off-target effects and reducing systemic toxicity [162].

#### 5.1.1. Polymer Conjugates

Attention to the field of polymer therapeutics has grown significantly over the last decade, along with advancements in the chemical synthesis and structural features of polymer–drug conjugates. A wide range of polymers, including N-(2-hydroxypropyl) methacrylamide (HPMA), poly(glycolic acid), poly(lactide-co-glycolide), and poly(ethylene glycol) (PEG) have all been utilized successfully in therapeutic applications.

Three distinct constituents have been used for the synthesis of these delivery systems: a medicinal drug, a targeting moiety, and a solubilizing unit [58,59,60,61,62]. The polymer backbone has these units covalently integrated into it. The conjugates’ water solubility is improved by the solubilizing unit. The targeting moiety facilitates more efficient conjugate delivery to the target cell or tissue.

Three synthetic methods are being used to create polymer–drug conjugates: adding a therapeutic agent to a synthetic polymeric carrier, adding a therapeutic agent to a monomer prior to polymerization, and adding a drug either as an initiator or monomer during the polymerization step [163].

It has been claimed that the issue of uncontrolled conjugation to the polymer backbone leading to high drug loading and controlled drug loading can be resolved by creating polymer–drug conjugates by incorporating a drug into a monomer prior to polymerization. The reaction of polymerization is not hampered by the drug’s conjugation to the monomer, and steric hindrance during conjugation is likewise resolved [164].

**Figure 4:**
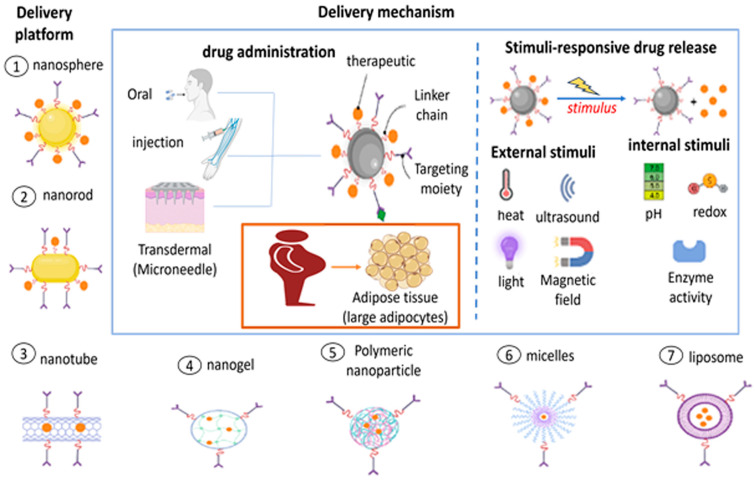
Key drug delivery mechanisms.

For the synthesis of polymer–drug conjugates, reactions of polymerization such as reversible addition–fragmentation transfer polymerization (RAFT), ring-opening polymerization (ROP), and ring-opening metathesis polymerization (ROMP) have been utilized, where the drug is first conjugated to the monomer [165,166,167]. Illustration of polymer drug conjugate is shown in Figure 4.

The conjugates made using the technique demonstrated a positive feature, including triggered drug release appropriate for conjugates packed with numerous medicines. A biodegradable backbone for polymer–drug conjugates has reportedly been produced by using ROP [168,169].

Polymer–drug conjugates have a number of benefits, including increased drug bioavailability and biodegradability [170], decreased drug toxicity [171], increased water solubility and drug stability, improved biocompatibility of the drug and delivery of the drug by maintaining and controlling the release mechanism of the drug [172], and the capacity to prevail the resistance of the drug. Moreover, there are a few restrictions on the use of polymer–drug conjugates in combination therapy, including challenges in determining the ratios of the integrated low loading capacity of the drugs and therapeutic agents [173].

In order to control appetite, the regulatory protein leptin is produced by adipocytes and crosses the blood–brain barrier (BBB). Nevertheless, leptin is often resistant to crossing the BBB because obesity impairs leptin receptor activity within hypothalamus and BBB transport [174]. Leptin has conjugated with amphiphilic Pluronic triblock copolymers to overcome this challenge. In order to enhance the pharmacokinetics (PK) of leptin in the peripheral body and its uptake in the brain, Yi et al. [175] proposed to alter leptin with Pluronic block copolymers. Pluronic is an amphiphilic triblock copolymer composed of poly(ethylene oxide)-b-poly(propylene oxide) b-poly(ethylene oxide) (PEO–PPO–PEO, same as poly(polyethylene glycol) b-poly(propylene glycol)-b-poly(polyethylene glycol), or PEG–PPG–PEG) [175].

Leptin has combined with Pluronic P85 at various random lysine amino groups or specifically at its N-terminal amine to further optimize the chemical formation of conjugates [176].

N-terminal conjugates with less obstruction to binding to the leptin receptor and low dosage were discovered to be transported more effectively to the brain and concentrated in the hypothalamus and hippocampus compared to native leptin and haphazard conjugates [177].

Chronic systemic inflammation has been associated with obesity in visceral adipose tissue (AT), and is initiated by pro-inflammatory macrophages [178]. However, at high doses, traditional treatments for inflammation can have severe adverse effects on tissues that are not the target of the treatment, such as liver cells, muscle cells, and fat cells. Precise targeting of macrophages within the adipose tissue (AT) surrounding internal organs (visceral AT) could lead to a substantial decrease in toxicity. Dexamethasone, a corticosteroid characterized by a half-life ranging from 36 to 72 h, interacts with the glucocorticoid receptor, leading to the suppression of pro-inflammatory gene transcription in M1 macrophages [179].

Due to the presence of dextran-binding C-type lectins and scavenger receptors, macrophages exhibit expression of these receptors, and dexamethasone–dextran compounds have been designed for selective uptake [180]. According to the findings, a significant proportion of the administered conjugates, specifically those conjugated with high-molecular-weight dextran (70 and 500 kDa), persisted in the visceral adipose tissue up to 24 h after the treatment, with a maximum retention rate of 63%. After esterase hydrolysis, the gradual release of conjugated dexamethasone resulted in its binding to the glucocorticoid receptor, where this binding process led to the inhibition of pro-inflammatory gene transcription in the adipose tissue (AT) of mice with obesity.

Despite the frequent utilization of natural polymers such as gelatine and chitosan as drug transporters, recent studies have suggested the potential for elevated levels of glycerol release from adipocytes treated with natural polymers, indicating a potential for lipolysis [181]. Because of their limited efficacy in delivery, natural polymers administered orally pose a notable drawback in the reduction of subcutaneous adipose tissue [182].

#### 5.1.2. Hydrogels

Hydrogels consist of water-soluble polymers that have been crosslinked to form a three-dimensional structure. Hydrogels possess the potential to function as drug carriers, thereby enabling spatiotemporal regulation of therapeutic release, and facilitating desirable drug delivery outcomes. The physicochemical properties of hydrogels are adjustable, and they can interact with biomolecules to regulate drug release and enhance therapeutic efficacy. Additionally, hydrogels can protect drugs from degradation and control degradability, thereby providing a versatile platform for drug delivery. Different-sized hydrogel particles can now be produced using a variety of manufacturing methods. The manufacturing parameters, such as flow rate, or the gelation conditions, such as the concentrations of the polymer and surfactant, can be used to adjust the dimensions of hydrogel [183].

##### Hydrogel Synthesis Methods

Polymerization and crosslinking are implied by the standard synthesis processes. These procedures can be carried out simultaneously in a single step or successively in a few phases [184]. The gelation process includes the polymerization step. The initial material’s structure and conformation have an impact on how soluble branching polymer networks develop [185]. Polymer monomers, prepolymers, or hydrophilic polymers are referred to as the beginning material [186]. In the creation of networks, the monomers and polyfunctional comonomers serve as crosslinkers. Due to their biocompatibility in aqueous environments [187], and primarily due to their capacity to load drugs [188], hydrophilic polymers are frequently employed to create hydrogels for the delivery of drugs. The structure of a hydrogel is determined by the hydration of the hydrophilic groups and domains present in the relevant polymers.

Since most bodily tissues are made up primarily of water, a hydrogel’s ability to swell is important for further usage in medical applications [189]. Different polymers’ swelling characteristics are beneficial for functionalizing with medicinal medicines. On the other hand, these systems’ efficacy might depend on their ability to administer these drugs without causing unwanted side effects.

In hydrogel swelling and degradation, the crosslinker agent is crucial [190]. It affects the final hydrogel product’s physical attributes [191]. By using crosslinking techniques, polymer monomers interact covalently or noncovalently to provide elastic properties [192]. This has led to the identification of two distinct categories of hydrogels; chemical gels are composed of networks formed by covalent bonds, whereas physical gels are created through noncovalent interactions [193]. On the other hand, there are elements that affect how hydrogels are assembled [194]. Permanent gels are produced in response to chemical stimuli, including pH [195], ionic strength [196], and solvent composition [197]. Temperature [198], the electric field [199], the magnetic field [200], light [201], and pressure [202] are physical stimuli that control the reversible phase transition, or change from an unswollen to a swollen state. Enzymes [203], antigens [204], and nucleic acids [205] are examples of biological stimuli that alter the hydrogels’ physical characteristics, such as solubility [206]. These have an impact on the hydrogels’ solubility and other physical characteristics.

Epigallocatechin gallate (EGCG), the predominant catechin present in green tea, exhibits a half-life duration between 1.9 and 4.6 h, and its effectiveness in inhibiting fat absorption has shown promising potential for treating obesity [207,208]. However, due to its low bioavailability, it is not suitable for clinical use. Zhang et al. [77] utilized poly(lactic-co-glycolic acid) (PLGA) to produce in situ hydrogel implants that contained EGCG. Furthermore, these implants were administered to mice that were induced to become obese through a high-fat diet (HFD) [209]. Over 30 days, the hydrogel-EGCG implant group reduced body weight gain by 35.6% compared to the control group. In addition, the administration of hydrogel-EGCG implants to mice resulted in decreased levels of total cholesterol, low-density lipoprotein (LDL) cholesterol, and triglyceride, while increasing the levels of high-density lipoprotein (HDL) cholesterol. These observations suggest that the use of in situ hydrogel implants could be a viable approach for the long-term management of hyperlipidaemia. The different utilized techniques for the characterization of hydrogels are listed in Table 4.

Hybrid hydrogels can be synthesized for the purpose of achieving controlled drug release. Liao et al. [210] loaded the protein hormone leptin into hydrogels composed of methylcellulose and gold nanoparticles. The proportion of gold NPs controlled the temperature-dependent degradation of hydrogels. As a result of adjustable light exposure, hydrogels discharged leptin, which collected in adipose tissue, consequently impeding adipocyte fat storage. In consideration of the positive results observed in vitro, further investigation is necessary to determine the feasibility of this hydrogel system’s responsiveness to stimuli in vivo.

An et al. [211] developed a disposable portable iontophoresis system. For drug delivery, the collaborative performance of a polypyrrole–polyvinyl alcohol-based conductive hydrogel system was investigated. The inclusion of electrically mobile drug nanocarriers (DNSs) within the polypyrrole–polyvinyl (PYP) hydrogel accelerates their mobility, resulting in improved drug delivery efficiency through iontophoresis. Additionally, the therapeutic potential of this system was evaluated in diet-induced type 2 diabetic and obese mice through transdermal delivery of ROSI via an electrically removable DNS. The system was applied using cathodic iontophoresis to the right inguinal region of obese mice. After four weeks of treatment, a significant reduction in blood glucose levels and a decrease in body weight by approximately 12% was observed. It was discovered that in the treatment group, there was a significant reduction in the size of AT. Histological examination revealed that there was significant browning at the site of administration. Finally, they performed a skin damage test and discovered that the system elicited neither skin irritation nor skin tissue inflammation.

**Table 4:** Techniques utilized for the assessment of hydrogel properties.

Characterization Technique	Abbreviation	Liposome Characteristics	References
Laser scanning confocal microscopy	LSCM	Pore dimensions and shape	[212]
Scanning electron microscopy	SEM	Morphological characterization, pore formation and pore size, and crosslinking status	[213]
Infrared spectroscopic analysis	FTIR	Chemical composition	[214]
	XRD	Phase behaviour	[214]
Differential scanning calorimetry	DSC	Thermal characteristics of hydrogels	[215]
Thermogravimetric analysis	TGA	Thermal stability	[215]
Atomic force microscopy	AFM	Topology and roughness	[216]
Swelling behaviour		To determine the swell-ability of these polymeric networks, the hydrogels are immersed in aqueous media or medium with a particular pH. These polymers exhibit swelling-related increases in dimensions	[217]

#### 5.1.3. Microneedles (MNs)

**Figure 5:**
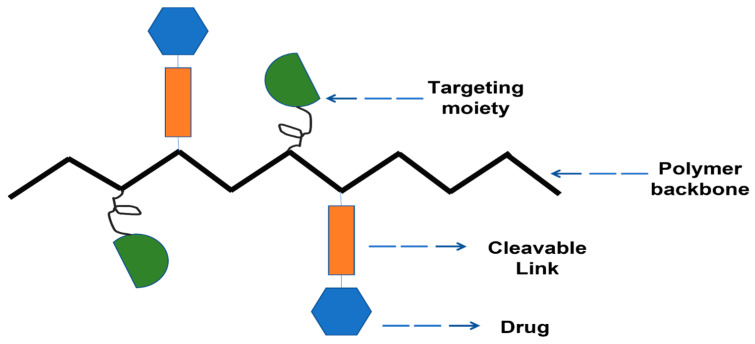
Schematic illustration of polymer–drug conjugate.

Microneedles (MNs), as a DDS, offer an alternative method of administration through surface skin, which was attempted in a clinic [218]. An MNs patch is composed of an array of tiny needles that range in height from 500 to 1500 µm. The MNs can penetrate the epidermal barrier to transport therapeutic agents in a minimally invasive way. The needles are constructed of biodegradable or water-soluble polymers that encase a drug, which is released at the insertion site as the needles dissolve or degrade. Transdermally delivered drugs’ local diffusion and accumulation allow for targeted delivery into subcutaneous AT while minimizing systemic side effects [219]. MN structures are frequently made of metal, silicon, or non-dissolving polymer and are utilized for poke-and-patch as well as coat-and-poke devices [218]. MNs are frequently manufactured via reactive ion etching [220]. Photolithographic technologies are commonly used in this process to set the dimensions of the base and the distance between MNs, as well as plasma chemicals, and they can be altered to adjust the shape of the MN as it converges into a sharp tip. This technology provides good control over MN shapes, although it frequently necessitates significant process adjustment. The technique of reactive ion etching is currently employed to fabricate MN arrays with ultra-short and sharp tips for delivering vaccines to the skin’s epidermal layer [221]. The common types of MNs are represented in Figure 5.

Also, wet etching of silicon was employed with photolithographic processes and silicon crystal planes defining the MN forms [222]. MNs projecting out of the substrate plane were commonly produced by silicon etching. Wet etching can also be used to create metal MNs, where the MNs were created in the plane of a metal sheet. This process results in the creation of linear or two-dimensional arrays through plane etching, followed by the bending of MNs at a 90-degree angle out of the plane [221]. MNs coated with medications, which have undergone clinical trials, are created using wet etching of metal sheets [223].

MNs have also been created using laser-cutting [224]. They are created from metal sheets in the same manner as wet etching, but the cutting is done with an Nd:YAG laser, which “draws” the shapes of the MNs without the need for a mask. Electropolishing is frequently required to remove rough edges from the laser-cut MNs. By “drilling” tapered holes into polymer sheets, laser ablation has also been utilized to create inverse moulds of MN arrays [225].

Polymer MNs are often manufactured by casting polymeric liquid solution onto an inverted mould of the MNs, which is frequently constructed of polydimethyl siloxane, to create dissolving MNs, hydrogel MNs, and, in certain situations, coated MNs [226]. The MNs are taken out of the mould when they are dried. Clinical trials have been conducted on MNs created in this manner for influenza vaccine [227]. Polymer MNs have also been created using two-photon polymerization [228].

In recent years, additive manufacturing technology utilizing a 3D printer has become increasingly prominent for the production of MN arrays [229]. This technology operates by depositing material layer by layer to build the intended structure. In recent times, the field of biomedical devices has witnessed remarkable progress in 3D printing technology, specifically for the generation of engineered tissue implants. Johnson et al. [230] designed the first MN master utilizing a commercial 3D printer in 2019.

Than et al. [77] created hyaluronic acid (HA)-based fast-dissolving MNs. In two minutes, the drug can enter the skin. They devised an animal experiment that delivered CL316243 and T3 to subcutaneous WAT in a short time. The MNs patch was applied to the groins of diet-induced obese (DIO) mice. Following a five-day treatment, MNs released CL316243, which was found to significantly induce the browning of white adipose tissue (WAT) cells and suppress weight gain. T3 reduced weight gain without causing systemic hyperthyroidism. The dose of the two drugs was lower when compared to the intraperitoneal injection group and there were almost no side effects noticed. There was no noticeable skin damage after administration, proving that the MNs patches were safe to utilize in mice. The food consumption of mice in the treatment group was nearly identical to that of mice in the control group. The treatment group exhibited a significant reduction in the weight of epididymal white adipose tissue (epiWAT) and inguinal white adipose tissue (igWAT) on the patch side. Additionally, there was a decrease in the total weight of igWAT on the non-patch side, albeit to a lesser extent [231]. These results suggest that the percutaneous administration of a browning agent can effectively treat regional adiposity and may also affect other adipose tissues via cutaneous circulation [77].

Research studies have demonstrated that caffeine, which is found naturally in coffee and tea, possesses properties that can resist obesity. Caffeine, which has been shown to reduce body weight by stimulating lipolysis, but has a low bioavailability that was attributed to its polymorphic transition from the anhydrous to hydrous form, was delivered using HA-based MNs [85]. The utilization of caffeine-loaded dissolvable HA MNs hindered the growth of crystals and caused a noteworthy increase in lipolysis, causing decreased levels of triglycerides, total cholesterol, and LDL cholesterol, resulting in a 12.8% weight loss in HFD-induced obese mice.

An et al. [62] recently reported dissolving MNs (DMNs)-mediated delivery of natural polymers for the treatment of obesity. Their findings revealed that the use of gelatine MNs, without incorporating any therapeutic agents, resulted in a 60% reduction of subcutaneous adipose tissue in rats with obesity induced by a high-fat diet through inducing lipolysis and inhibiting lipogenesis. This effect could be mediated by glycine, which accounts for 30% of the amino acids in gelatine and has previously been shown to lower adipose tissue and total body weight [232].

MNs also have the potential to serve as an efficient carrier for the delivery of nanoparticles (NPs) into the skin’s intradermal layer, with microconduits in the epidermis acting as conduits facilitating the entry of NPs into therapeutic sites. Zhang et al. [78] created a transcutaneous patch with polymeric MNs to deliver anti-obesity therapeutics locally and induce AT transformation. To achieve a prolonged release of browning agents CL 316243 or rosiglitazone, researchers utilized pH-responsive acetal-modified dextran nanoparticles as a carrier. These drug-loaded nanoparticles were subsequently incorporated into a crosslinked hyaluronic acid-based MN array, facilitating skin penetration and targeted delivery to the inguinal adipose tissue while restricting systemic exposure. pH-sensitive NPs gradually degraded under physiological glucose conditions, releasing the agent into the AT and promoting browning. Studies conducted on mice with high-fat diet (HFD)-induced obesity demonstrated that the formation of MNs in vivo enhanced systemic energy expenditure and increased fatty acid oxidation. Moreover, it also improved insulin sensitivity, and resulted in a 15% reduction in weight gain.

Zhang et al. [151] developed a percutaneous DMNs patch that delivers caffeine through the skin and observed its anti-obesity effect in DIO mice. Oral caffeine has limited availability, and its blood concentration decreases rapidly upon administration. However, delivering caffeine through the skin is challenging due to crystal growth caused by the multiform transition from anhydrous to aqueous conditions. To overcome this, the authors created a caffeine-delivering DMN based on HA that keeps caffeine anhydrous and inhibits crystal growth. DIO mice were treated with DMN three times a week for six weeks, resulting in a significant decrease in body weight by approximately 13%. The food intake of obese mice in each group did not change significantly. Furthermore, serum triglyceride and total cholesterol levels, as well as other biochemical indicators of obesity, decreased significantly in DIO mice, confirming the anti-obesity effect of the system [151].

The frequent and long-term administration of anti-obesity drugs is required for managing obesity. Consequently, the development of sustained-release anti-obesity medications holds great promise. Yang et al. [233] formulated slowly dissolving MNs patches using PLGA (poly(lactic-co-glycolic acid)) and Cy5 fluorescent molecules. The drug delivery system demonstrated a sustained-release effect, as evidenced by the retention of a fluorescence signal at the injection site for up to five days. The efficacy of the MNs patches was tested by treating diet-induced obese mice with sustained-release patches containing CL316243, resulting in a 15% reduction in weight gain compared to non-drug patches. However, intraperitoneal injections of the same dose of CL316243 did not result in significant weight reduction. Transdermal delivery of CL316243 using MNs increased body temperature and UCP1 (uncoupling protein 1) expression in adipose tissue, which confirmed the promotion of browning. There were no significant differences in food intake or skin abnormalities between the groups. Additionally, MNs treatment led to a decrease in metabolic syndrome indicators, such as total cholesterol, free fatty acids, and insulin, as revealed by serum biochemical indexes in mice [233].

Zhang et al. [78] developed a patch consisting of nanoparticles (NPs) and MNs that can deliver two browning agents, ROSI or CL 316243, to the groins of mice. They first tested the patch on lean mice and found that the groups treated with ROSI-NPs-MNs or CL316243-NPs-MNs had increased numbers of beige adipocytes, upregulated UCP1 gene expression, and downregulated IL-6 gene expression in their inguinal adipose tissue (AT) compared to the HA-MNs patch group. They also observed no significant differences in food intake or oxygen consumption between the groups. Next, they applied the patches to obese DIO mice and found that the treatment group had a 15% reduction in weight gain and a 30% reduction in epididymal white adipose tissue (epiWAT).

Yixuan Xie et al. [234] developed a biodegradable MNs patch made of PLGA and PLA and investigated the effect of CL316243 MNs patches on DIO mice. The treatment group had a weight loss effect and an increase in brown adipose tissue (BAT) weight, with a decrease in inguinal white adipose tissue (igWAT) weight, and the expression of UCP1 in the treatment group was also significantly increased. Notably, the dose in the MNs patch was one-tenth that of the injection dose, but the therapeutic effect was just as strong.

The transdermal method of insulin delivery for managing diabetes on a daily basis is less invasive and more patient-friendly compared to the conventional hypodermic injection. In recent times, MN techniques have surfaced as an alternate approach to administering drugs through the skin. These tiny needles can easily penetrate the outermost layer of the skin, the stratum corneum, without causing any pain and can access the epidermal and dermal layers to release drugs. Novel MNs have been created that can react to changes in glucose levels in the body to release insulin as and when required.

Martanto et al. [235] showed that insulin can lower blood sugar levels in diabetic rats by using MNs. They created an array of 105 tiny needles by cutting stainless steel sheets with a laser, and then inserted them into the skin of the rats. Afterward, they applied an insulin solution to the skin and left it in place for 4 h. These MNs facilitated the delivery of insulin through the skin, resulting in a decrease in blood glucose levels of up to 80% in vivo.

Liu et al. [236] created HA-based MNs using micromoulding techniques and studied their effectiveness in delivering insulin through the skin. The insulin that was loaded onto the MNs remained over 90% bioactive, even after being stored for a month at various temperatures. Additionally, the HA MNs proved to be more resistant to humidity-induced deformation than sugar glass MNs. In animal studies on diabetic rats, the HA MNs loaded with insulin demonstrated a hypoglycaemic effect that varied depending on the dose administered. The temporary microchannel created by the insertion of the MNs disappeared within 24 h. In addition, the application of MNs and iontophoresis together was investigated to expand the variety of drugs that can be delivered through the skin [237].

Chen et al. [238] demonstrated that insulin absorption from nanovesicles was significantly higher when driven by iontophoresis through microchannels induced by MNs compared to passive diffusion. Specifically, they found a 700-fold increase in absorbance rate. The nanovesicles with a positive charge demonstrated remarkable permeability when combined with MNs and iontophoresis. As a result, they were able to lower the blood glucose levels in diabetic rats by 33.3% and 28.3% from the initial levels after 4 and 6 h, respectively.

A study using a tip-dissolvable MN array containing insulin to treat STZ-induced type 1 diabetic SD rats showed that the tip-dissolvable MA was found to be an effective method of delivering insulin in vivo, as it was able to maintain blood glucose levels at a normal range for an average of 3.4 ± 0.5 h, compared to only 1.6 ± 0.4 h for subcutaneous injection. Thus, the tip-dissolvable MA could be a viable alternative for transdermal drug delivery [239].

Furthermore, the effectiveness of MNs for drug delivery has also been studied in humans. In a study by Gupta et al., the transdermal delivery of insulin using hollow metal MNs was tested on adults with type 1 diabetes [240].

Additional trials are currently being conducted to assess how safe and effective the use of MNs is for delivering insulin to humans. An insulin pump was attached to MNs, and placed on the skin of the abdomen in order to regulate the rate at which insulin is delivered. The findings indicated that when the MNs were inserted to a depth of 1 mm into the skin, insulin was absorbed quickly, and blood glucose levels decreased [241].

During MN design, mechanical testing such as axial force, transverse force, base plate break, and insertion force should be applied to characterize the MN’s mechanical properties to ensure that it can withstand epidural insertion without failure [230].

Several techniques utilized to assess MNs, such as axial force and transverse and insertion forces and their descriptions and indications are listed in Table 5.

#### 5.1.4. Micro- and Nanoparticles

**Figure 6:**
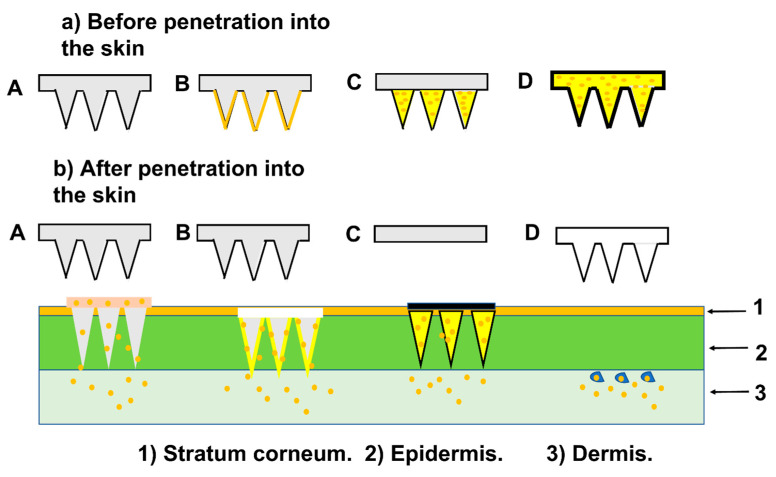
Types of MNs. (A) Solid MNs are employed for skin preparation and use a poke-with-patch technique. (B) Coated MNs employ a coat-and-poke strategy, with a medication solution coating applied to the needle’s surface. (C) Dissolving MNs: biodegradable polymers are used to make dissolving MNs. (D) Hollow MNs: the medication solution is loaded into hollow MNs, which deposit the drug in the dermis.

In contrast to commonly used MNs for drug delivery, particulate drug delivery systems offer several advantages. Particles such as hydrogel implants and MNs enable direct accumulation at the treatment site, resulting in high local drug concentrations and minimal systemic toxicity. Additionally, particles can serve as reservoirs for slow drug release, allowing for a more systematic effect. Moreover, particles can be administered systemically and be targeted to specific locations through active or passive targeting approaches, making their use a versatile drug delivery method [244]. The release of active molecules from micro- and nanoparticles may follow various mechanisms depending on their design, as can be observed in Figure 6.

A distinction is frequently made between microparticles and NPs, which are particles with dimensions that are within the ranges of micrometres and nanometres. The dissimilarity in particle size has a significant impact on numerous factors, ranging from in vitro traits to in vivo applications. Capsaicinoids have been shown to increase energy expenditure by 50 kcal/day, resulting in clinically significant weight loss in 1–2 years [245]. Capsaicin was encapsulated into polycaprolactone (PCL) microparticles by Almeida et al. [246]. PCL microparticles were utilized to achieve a controlled and gradual release of capsaicin, with no change in its biexponential release kinetics. The optimized particulate formulation effectively improved capsaicin’s gastric tolerability by preventing inflammation in the stomach’s submucosal layer and decreased mesenteric and retroperitoneal fat deposits in obese rats.

Researchers have created microspheres using chitosan, which are loaded with capsaicin, and evaluated their impact against obesity. The study was conducted by administering the microspheres orally to rats with diet-induced obesity [247]. Capsaicin-encapsulated microspheres outperformed native capsaicin and the commercial agent Orlistat in terms of controlling body weight, body fat, and serum lipids.

One promising method for achieving site-specificity is a local injection into the target tissue. Microparticles have also been used for obesity treatment via local injections due to their favourable properties for avoiding rapid drug diffusion and extending drug local retention. Lucas et al. [248] created microparticles of human serum albumin–alginate-encapsulating melanocyte-stimulating hormone (MSH), an anorexigenic neuropeptide with anti-obesity properties.

It was discovered that the controlled release of melanocyte-stimulating hormone (MSH) in the hypothalamus can be achieved by administering microparticles through hypothalamic injections [249]. This technique enables the specific targeting of the paraventricular nucleus while preventing the degradation of the peptide. In comparison to the control and native MSH groups, rats treated with MSH-loaded particles showed a consistent reduction in body weight gain over an extended period.

PLGA microparticles have been utilized in AT to locally suppress the Notch signalling pathway [155]. Notch signalling has been shown in the past to promote adipocyte browning and improve energy metabolism [250,251]. Although dibenzoazepine (DBZ) is a well-known inhibitor of Notch signalling, systemic Notch inhibition may cause off-target toxicity in the gastrointestinal tract [252]. The study examined the impact of DBZ-loaded PLGA microparticles on the induction of browning in white adipose tissue (WAT) in lean mice. The results demonstrated that the DBZ released from the microparticles maintained its bioactivity after being injected locally into the inguinal WAT, and efficiently stimulated the browning of white adipocytes by inhibiting Notch signalling. Importantly, the localized release of DBZ in the inguinal WAT reduced the potential adverse effects of systemic administration. Microparticles offer potential advantages for controlled drug delivery by allowing for a high concentration of the drug to be administered locally over an extended period [252].

Microparticles are unlikely to cross most biological membranes because of their larger size. They can also cause acute and chronic inflammatory responses due to the slow degradation of particulate materials. Nanoparticles (NPs) address some of the limitations of microparticles and offer additional benefits, such as a high surface-to-volume ratio, customizable surface chemistry, and intracellular drug release. These advantages make them a hopeful delivery system for treating diseases, such as obesity [253].

There are two types of nanotechnology methods for the preparation of the particles: attrition and precipitation [254,255]. In addition to their beneficial small-size stability, emulsions are simply and cheaply manufactured. They can also be specially tailored to deliver larger concentrations of medicinal substances to targeted regions. Emulsions are, therefore, very good options for therapeutic treatments against specific diseases such as obesity. Nano-emulsions that are colloidal particles dispersed in oil-in-water (O/W) or water-in-oil (W/O) dispersions, with emulsifying agents acting as surfactants, which provide thermodynamic stability [256], utilize lipids that come from one of the components of cell membranes. They have a tendency to merge with cells without discrimination during circulation throughout the body [257]. This non-specificity can be avoided by adding poly(ethylene glycol) (PEG) to their surface, which causes a “stealth” feature with limited or no uptake by the reticuloendothelial system [258].

Gold NPs (AuNPs) are effective anti-obesity drug carriers due to their ability to absorb visible and near-infrared (NIR) light, small size, large surface area, and ability to be functionalized with various molecules, which make them ideal for drug delivery systems. In the context of obesity treatment, AuNPs can be used to deliver anti-obesity drugs directly to the target tissues [259]. One approach is to functionalize the surface of AuNPs with specific ligands that can bind to receptors on adipocytes, the cells responsible for fat storage. By targeting these receptors, AuNPs can deliver anti-obesity drugs directly to adipose tissue, allowing for a more targeted and efficient treatment. This approach minimizes off-target effects and enhances drug efficacy [260].

Additionally, AuNPs have been used in combination with photothermal therapy for obesity treatment. Photothermal therapy involves using near-infrared light to heat up gold nanoparticles, which then generate heat and cause localized damage to adipose tissue. This localized damage can lead to the shrinkage of fat cells and a reduction in body fat [261].

Lee et al. [86] created hyaluronate–hollow gold nanosphere–adipocyte targeting peptide (HA-HAuNS-ATP) conjugates for photothermal lipid decomposition. HA can improve HAuNS stability and biocompatibility. The photothermal properties of HAuNS are superior. ATP with specific sequences can improve the system’s targeting of AT. Cytotoxicity tests showed that the system will not cause significant cell damage. Photoacoustic imaging (PA imaging) results showed that the conjugation of HA-HAuNS-ATP was found to be highly effective in penetrating the abdominal skin of mice through the transdermal route. The researchers utilized conjugates to treat mice that were obese and fed with a high-fat diet. They then observed the decomposition of fat through photothermal means [262]. The findings demonstrated that when subjected to NIR radiation, the HA-HAuNS-ATP conjugates were more efficient in breaking down the adipose tissue of obese mice, leading to a reduction in fat content of approximately 20%. Furthermore, the conjugates did not cause any damage to the skin [86].

Despite the potential effectiveness of using AuNPs in treating obesity, it is vital to recognize that further investigation is required to gain a complete understanding of their safety and efficacy. Examining the long-term impacts and possible side effects of AuNPs in humans mandates animal studies and clinical trials. Nevertheless, AuNPs offer a thrilling prospect in the creation of new and precise therapies for obesity treatment.

#### 5.1.5. Liposomes

Liposomes, which are sphere-shaped vesicles made up of one or more concentric lipid bilayers, are another drug delivery vehicle [263]. These carriers provide a non-selective combination or merging with cells while circulating throughout the body and can be employed to enable contact-based transport through the transfer of lipids between the cell membrane and the lipid layer of liposomes [264]. Typically, liposome production involves two primary phases: drying out lipids from an organic solvent and dispersing them in a water-based solution.

In the thin-layer hydration technique, the mixture of lipids that give the liposomes a surface charge is typically dissolved in chloroform [265], or it is mixed with a polar solvent, most frequently methanol [266], in order to create the liposomes. A thin coating of lipids is then created once the solvents have evaporated. As an alternative, the evaporation process might take place in an environment of inert gases such as nitrogen or argon [267]. After that, the film undergoes exposure to water, a buffer (such as a phosphate buffer adjusted to the appropriate pH), or an aqueous solution that holds the hydrophilic active ingredient, which will be enclosed inside the liposomes. This process results in the production of a solution containing MLV liposomes with a broad range of sizes.

Different methods, such as sonication [268] and extrusion [269], are employed to standardize the size and decrease the diameters of the liposomes. Before the thin film is created, the remaining lipids are combined with the lipophilic components inserted into the lipid bilayer. The technique makes it possible for both hydrophilic and hydrophobic compounds to be trapped at the same time. The lipid-soluble part is dissolved in the lipids, and when the liquid evaporates, it creates a thin layer of lipids. To add moisture to the layer, the hydrophilic active ingredient is dissolved in a water-based solution [270].

The thin-film hydration technique has been updated to become reverse-phase evaporation (RPE). In this instance, an aqueous phase is combined with lipids that have been dissolved in an appropriate solvent. Afterward, the unstable organic solvent is evaporated at a temperature higher than its phase transition point. This process is repeated by adding more water phase and evaporating it again until all the organic solvents have been eliminated [271]. The subsequent treatment of the unprocessed liposomes acquired by the reverse-phase evaporation approach is the same as that applied with the TFH method. The resultant liposome dispersions are next homogenized to dilute them and improve the uniformity of the heterogeneous vesicles.

The injection of ethanol is another widely used technique to produce tiny liposomes. In the case of this procedure, pure water, a buffer solution with a specified pH, or a solution of an active substance that is hydrophilic is prepared [272]. The ethanol solution of lipids contains a lipophilic medication. For better consistency of the results, the injection of the ethanol solution can be done either manually using a syringe or automatically with a pump.

During the injection process, the hydrophilic phase is heated to a temperature higher than its phase transition temperature beforehand. A vacuum rotary evaporator is used to evaporate the ethanol after the carriers have been acquired [273]. The injection approach has a variety of benefits, including simplicity, equipment independence, and the avoidance of poisonous and dangerous solvents like methanol or chloroform. Furthermore, with proper control of the process variables (such as the lipid phase composition, auxiliary surfactants, or the addition of cholesterol), it is possible to produce liposomes with such a restricted size range without the use of additional homogenization methods, such as sonication or extrusion [274].

This method’s drawbacks include the requirement to remove the ethanol from the mixture and the limited encapsulation efficiency when the injection is made into a large volume of water in the case of excessive lipid dilution [275]. An approach called pre-concentration is utilized to achieve higher loading rates. In this instance, a tiny portion of the aqueous phase is injected with ethanol before the remainder is added after the alcohol has evaporated [274].

In the procedure for the detergent removal method, a suitable organic solvent is used to dissolve lipids along with a high critical micelle concentration (CMC) surfactant in a round bottom flask. After mild solvent evaporation, a thin coating is produced at the flask’s bottom [276]. The lipid film is subsequently hydrated in an aqueous solution that contains drug molecules to produce a mixed micelles solution [277]. The surfactant can be eliminated through one of the following techniques: dilution, size-exclusion chromatography, adsorption onto hydrophobic beads, dialysis, or any combination of these techniques [278,279,280,281]. After solution concentration, an LUVs liposomes vesicle will be created [282]. The separation of the majority of hydrophilic medications from the liposomes during the detergent removal stage is a major flaw in this approach [283].

**Figure 7:**
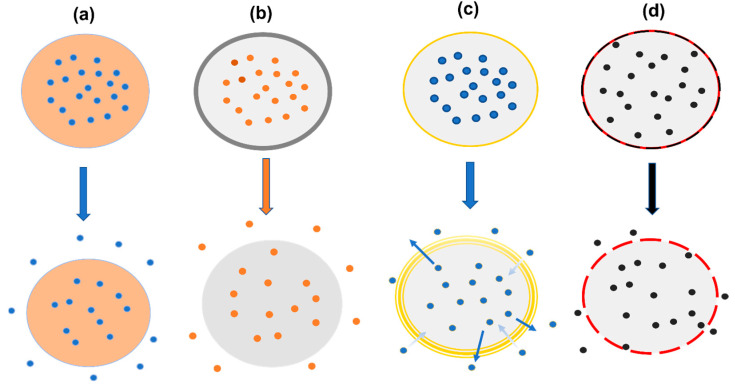
Release behaviour of drug-encapsulated microparticles: (**a**) diffusion through polymer network, (**b**) dissolved coat layer, (**c**) permeable membrane, (**d**) semipermeable porous coat.

Liposomal drug delivery through systemic administration can be problematic due to non-specific uptake by cells and unintentional entrapment in non-targeted organs. However, the surface of liposomes can be modified with hydrophilic polymers like PEG to minimize uptake by the reticuloendothelial system, reduce renal clearance, and prolong circulation time [264]. As a result, the medication can be more effectively maintained within the body and produce its intended healing effects. Therapeutic delivery system examples composed of liposomes are shown in Figure 7.

**Figure 8:**
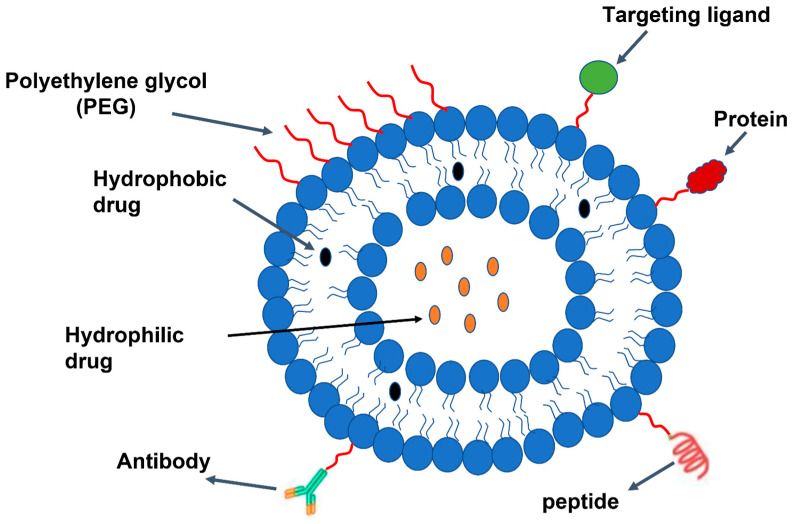
Generic structure of liposome-based therapeutics delivery systems.

Several functionalized liposomes have been created with ligands to improve the effectiveness of drug delivery to adipose tissue, and they are considered to be safer and more specific. Hossen et al. [284,285] altered the surface of liposomes using PEG and a circular peptide (KGGRAKD), which attaches specifically to the endothelial cell-surface prohibition in white adipose vessels (Figure 8). The liposomes were taken up by primary endothelial cells through prohibition-mediated endocytosis and were able to escape endosomes and lysosomes. When administered intravenously to lean mice, PEGylated targeted liposomes accumulated more in white adipose vessels than non-PEGylated targeted liposomes. Additionally, there was a significant reduction in the undesired accumulation of particles in the liver.

Furthermore, in obese mice, liposomes that were targeted specifically accumulated in adipose vessels and clusters of angiogenic adipocytes after systemic administration. Interestingly, non-targeted liposomes that were PEGylated were also present in these clusters due to an unexpected passive targeting mechanism, which may have been due to increased tissue permeability and retention. In subsequent research, anti-obesity drugs like proapoptotic peptide D(KLAKLAK)2 and cytochrome C, which initiates apoptosis, were enclosed in prohibition-targeted nanoparticles [286,287]. In obese mice that were given a high-fat diet, administering targeted nanoparticles through the body significantly decreased the gain in body weight, levels of leptin in the blood, and deposits of fat in the liver and muscle. Additionally, there were no signs of liver damage related to the use of these nanoparticles, indicating that the composition of the liposomes used was both safe and biocompatible. When evaluating the physiochemical properties of liposomes, parameters such as average size, size distribution, surface charge, shape, morphology, lamellarity, encapsulation efficiency, phase behaviour, and in vitro release profile are all taken into consideration. Most of the available liposomes need to be characterized using several techniques, listed in Table 6, to figure out their physicochemical, morphological, and topographical features and determine their possible applications.

### 5.2. Drug Loading and In Vitro Drug Release Profile from Drug Delivery

To generate a therapeutic effect, conventional drug administration frequently requires high dosages or repeated administration. This can lead to reduced effectiveness and lower patient adherence, as well as potentially causing significant side effects and toxicity. Oral administration, the most common method of drug delivery, is typically limited by inadequate targeting and brief circulation periods lasting only around 12 h [293]. Peptide and protein-based medications often remain active in the bloodstream for a short period, typically ranging from a few minutes to several hours [294]. To overcome these difficulties, recent decades of study have concentrated on controlled DDSs that can control how medications are delivered to cells and tissues throughout time and place. They can, in theory, leverage therapeutic benefits by increasing efficacy while decreasing toxicity and required dosage.

#### 5.2.1. Polymer Conjugate

Polymer conjugates refer to a type of drug carrier that links a bioactive drug molecule and a polymer carrier through a covalent bond. The drug molecule is deliberately designed to be conjugated to the polymer carrier, and as the polymer carrier degrades over time or in response to certain triggers, the drug is released. The conjugated drug remains biologically active and acts as a structural element in the assemblies. Compared to conventional therapeutics, polymer conjugates have superior performance due to their ability to be fine-tuned for drug release and the increased stability of the native drugs.

A new approach to enhancing the effectiveness of appetite control and thermogenesis induction delivery involves using polymer conjugates that can penetrate biological membranes.

Typically, a linker helps the medicinal drug integrate into the polymeric backbone [295]. The release of the medication that is linked to another molecule depends on certain conditions, like alterations in pH, the presence of enzymes, or the susceptibility of specific diseased organs or tissues. In such situations, the linker assumes a crucial role [296].

The duration of action of medications with slow renal clearance and inert metabolism is prolonged. The conjugation results in a delayed renal excretion, increased blood flow, and an endocytotic cell uptake [297]. The kind of linkers employed affect how much of the medication is loaded onto the carrier, how stable the drug is, and how it releases from the carrier.

Celastrol was combined with carboxymethyl chitosan to form the celastrol conjugate (Cel-CMCS), which has great solubility in water (33.94 mg/mL), and its anti-obesity activity was then studied in diet-induced obese mice.

According to the UV spectra results, it was found that Cel-CMCS had a 1.5 wt% celastrol content. Moreover, the water solubility of Cel-CMCS was significantly higher than the natural celastrol. In addition, the in vitro stability analysis demonstrated that Cel-CMCS had controlled release properties for celastrol in both PBS (pH 7.4) and SIF (simulated intestinal fluid, pH 7.5) [296].

#### 5.2.2. Hydrogel

Hydrogels have the ability to manage the distribution of various therapeutic substances, such as small drug molecules, large molecular pharmaceuticals, and cells, both spatially and temporally [298]. They act as a foundation for various physiochemical reactions with enclosed medications, and can regulate drug delivery thanks to their customizable physical characteristics, manageable degradability, and capacity to protect delicate compounds from deterioration [183].

How the hydrogel releases the drug is often crucial in achieving the desired therapeutic effects. The appropriate duration of drug availability (short-term or long-term) and the type of drug release (continuous or pulsatile) depend on the specific use case. Hydrogels consist of a polymer network that is crosslinked and has spaces between the polymer chains known as “meshes”. These spaces allow for the transportation of liquids and small solutes. The typical size of the mesh in hydrogels varies from 5 to 100 nm, and there are many techniques available for determining its size. Because it regulates steric interactions between the medications and the polymer network, the mesh size determines how drugs diffuse through a hydrogel. When the pores are larger than the drug, diffusion takes over the drug release mechanism. The network does not significantly affect the diffusion of small drug molecules, regardless of the mesh size.

Controlling the release of medicinal molecules that were initially enclosed in a hydrogel can be achieved by regulating the degradation of the network. As the network of the hydrogel degrades, its mesh size increases, which enables medications to diffuse out. This degradation process can occur through hydrolysis [299], or enzyme activity [300], and typically takes place in the polymer backbone or at the crosslinks.

Hydrogels’ controlled swelling is a second technique for releasing entrapped medicines. As a hydrogel swells, its mesh size increases. The extent to which a hydrogel swells is controlled by a delicate balance between the forces that restrict network distortion and the process of osmosis, which causes the absorption of water [301]. Swelling behaviour can be affected by a variety of extrinsic factors, including temperature, glucose, pH, ionic strength, and electric and light fields [183].

One disadvantage of the controlled swelling of systems is that the response time for macroscopic hydrogels is somewhat slow due to the slow diffusion of water. Another way to free drug molecules from their confinement is by mechanically distorting the network structure. This can lead to two effects: the mesh size can expand through alterations in the network’s topology, and there can be movement of fluids inside the network due to convective flow [302].

#### 5.2.3. MNs

The design of MN arrays, including their form, size, geometry, manufacturing materials, and procedures, is crucial in addressing critical issues in medication delivery. The efficacy of MNA-based drug delivery is impacted by the type of active substance supplied. There are different strategies involved, such as “poke and patch”, “poke and flow”, “coat and poke”, and “poke and release” [218].

The “poke and patch” technique involves the use of solid MNs to puncture the skin and create microchannels that penetrate the deepest layers of the epidermis. By breaking down the principal barrier to permeability, the stratum corneum, this method dramatically improves passive diffusion through the skin [303].

One example of this technique is drug-coated MNs, wherein the drug is coated on solid MNs before being inserted into the skin. Once the coated MNs are introduced into the skin, the drug is distributed into the systemic circulation. This method is suitable for transporting complex and large molecules, including deoxyribonucleic acid, medications such as desmopressin, and parathyroid hormone. After the insertion of MNs into the skin, this technique enables the drug to diffuse from the coated surface to the deepest epidermal layer [304]. MNs were utilized through different approaches to deliver active molecules, as listed in Table 7.

To administer drugs through poke and release, MNs rely on biocompatible polymers like polyvinyl alcohol, carboxymethyl cellulose, or sugars [177]. These polymers are inserted into the skin and gradually dissolve, releasing the drug into the bloodstream in a controlled manner. The drug is released by either the needle or the coating on the needle dissolving [305].

In this process, the hollow bore channel in the needle enables the drug to enter the body through the skin, where it diffuses into the systemic circulation [306].

#### 5.2.4. NPs

Treating obesity through systemic administration is an effective approach. Nanotechnology utilizes PEGylation to shield substances from the host’s immune system, which prolongs their circulation by decreasing renal clearance in the body. This protection enables the medicine to infiltrate and remain in bodily tissue, enhancing its therapeutic efficacy by delivering it to the disease site [307].

Mascolo et al. [308] created rosiglitazone-encapsulated PLGA NPs enclosed by a steric-repellent PVA outer layer. This technique decreases NP aggregation and diminishes protein modification and opsonization in the blood after intravascular injection. NP treatment for HFD-fed Ldlr -/- mice resulted in a displayed reduction in inflammatory macrophages in the WAT and liver without the adverse effects on lipid metabolism or cardiac tissues that are commonly seen with native rosiglitazone administration [169].

Efficient drug delivery to specific therapeutic targets is always challenging. To achieve this, NPs can be altered with surface ligands, targeting molecules, or peptides. Because angiogenic factors stimulate AT crosstalk and angiogenesis stimulation results in the browning of AT [309,310], vasculature-directed targeting of AT may be a potential therapeutic intervention for obesity.

Xue et al. [311] encapsulated rosiglitazone or a prostaglandin E2 analogue in PLGA-b-poly(ethylene glycol) (PEG) NPs and modified the NP surface with adipose vasculature-targeted peptides iRGD (CRGDK/RGPD/EC) or P3 (CKGGRAKDC), which bind to specific antigens. When compared to free drugs and non-targeted NPs, more-targeted NPs were able to localize in the AT, releasing drugs following intravenous injection. In HFD-induced obese mice, this approach increased the browning of WAT and inhibited body weight gain.

Drug delivery to the mitochondria is generally difficult due to AT mitochondrial dysfunction, linked to obesity. However, Marrache et al. [312] created mitochondria-targeting polymeric NPs by combining a targeted PLGA-b-PEG-TPP polymer with either non-targeted PLGA-b-PEG-OH or PLGA-COOH. Because of their high buffering capacity, targeted NPs achieved high endolysosomal capability and mitochondrial uptake efficacy. Moreover, it is possible to adjust the effectiveness of the NPs by modifying their surface charge and size. In addition, targeted NPs containing the mitochondrial uncoupler 2,4-DNP were found to inhibit the differentiation of 3T3-L1 preadipocytes at a lower dose compared to the free drug [169].

Researchers have recently explored the use of local drug delivery for the treatment of obesity, as it can help maintain drug levels and reduce the required dosage. Jiang et al. [313] encapsulated DBZ in PLGA NPs and administered them directly into the inguinal WAT of HFD-induced obese mice. This approach stimulated the browning of white adipocytes, improved glucose homeostasis, and reduced body weight gain by blocking the Notch signalling pathway.

Notably, adipocytes showed a preference for endocytosing NPs following local administration, which restricted the distribution of the therapeutic agent in the body and minimized its adverse effects on non-adipocyte cells. As a result of enhanced intracellular transportation, NPs loaded with DBZ effectively impeded Notch signalling using a dosage that was fifty times lower than that of intraperitoneal injection and ten times lower than that of microparticles. NPs delivered different medications by both injection and oral delivery for the treatment of obesity and its complications, as can be noted from Table 8.

Nanofibers increase the rate of drug diffusion and uniform distribution on the skin’s surface [314]. Ariamoghaddam et al. [80] created a DDS of polyvinyl alcohol–gelatine nanofibers with diameters of 200–250 nm, which were used to deliver curcumin to treat rats with obesity. Curcumin is a substance derived from common edible fungi that are traditionally believed to provide various advantages such as weight loss, anti-inflammatory properties, anti-cancer effects, and more [315]. Browning may be responsible for curcumin’s anti-obesity effect, according to some studies [79]. After 20 h, the curcumin release rate increased to more than 50%. One possible explanation for the fast spread of curcumin could be its combination with gelatine, which enhances its solubility and leads to more effective utilization. By administering curcumin in this way, whole-body magnetic resonance imaging has suggested that the overall amount of adipose tissue in rats decreases by 4% to 7%.

**Table 8:** Use of NPs in the treatment of obesity and related diseases.

Delivery Method of NPs	Materials	Disease	Reference
Injection	PLGA	Obesity	[313]
	Egg yolk phosphatidylcholine (EPC), cholesterol, and peptide-conjugated PEG-lipid		[287]
	PLGA and PEG		[311]
	Egg yolk phosphatidylcholine, PEG, and peptide		[285]
	MSN and PCL		[316]
	PLGA and PVA	Obesity and inflammation	[308]
	LITA	Obesity and liver lipid accumulation	[317]
	YSK05, cholesterol and mPEG-DMG	Type 2 diabetes	[318]
	Oligopeptide (ATS-9R)		[319]
	Zinc oxide		[320]
	Egg yolk phosphatidylcholine and cholesterol		[286]
	Dextran	Type 2 diabetes and heart disease	[180]
	PLGA	Heart disease	[321]
	CHC	Diabetes	[322]
Oral	GLP-1	Type 2 diabetes	[323]
	Chitosan, gamma-PGA	Diabetes	[324]

#### 5.2.5. Liposomes

Both passive and active methods can be used to load drugs into liposomes. While the lipid bilayer is forming, passive loading traps hydrophilic medications in the aqueous core of the liposomes, while hydrophobic pharmaceuticals build up in the tiny hydrophobic lipid bilayer [325]. Bilayer instability, rapid drug release, and a high drug/lipid ratio are all problems with passive loading [326].

Therefore, by constructing a drug-in-cyclodextrins-in-liposomes delivery system, researchers were successful in increasing the water solubility of these hydrophobic pharmaceuticals. This allowed for liposomes to be loaded with an aqueous core. To ensure excellent encapsulation efficiency of priceless chemotherapeutic drugs, active or remote loading has been established [327]. It is possible to load preformed liposomes remotely by creating a pH gradient or ionic differences across the bilayer membranes of the liposomes. The drug’s aqueous solubility and the existence of an ionizable functional group in the chemical make up the two key factors that determine the efficacy of intraliposomal remote loading. It was created to actively load hydrophobic medicines into liposomes in reaction to ionic and/or pH gradients across the liposomes’ bilayer [328].

Hydrophobic medications can be made to accumulate inside the core of liposomes using this process once the vesicles are made. The benefit of this approach is that the drug loading can be done without regard to the circumstances surrounding liposome production. The majority of potentially active substances are weak bases that can be loaded in reaction to pH gradients and contain amine functional groups, which can be primary, secondary, or tertiary [328]. Drugs that lack an ionizable functional group or are not weak bases can be transformed into prodrugs that have low basicity or are enclosed within carriers modified with amino groups like cyclodextrins to enable encapsulation and intraliposomal retention [329]. A specified molecular weight is removed from the liposomal sample before it is hermetically sealed inside the dialysis bag. The tubing membrane system is placed in a buffered saline solution with a pH of 7.4 to replicate natural fluid methods of release. To simulate an in vivo environment, the entire system is maintained at 37 °C while being constantly stirred. An aliquot of the sample is obtained at predetermined time intervals, and it is examined using the standard techniques for drug quantification. The number of samples must remain consistent. Consequently, an equivalent volume of new release medium is added once more to the system [330,331]. The release profile is generated using data by plotting the cumulative release percentage against specific time points. This in vitro release study is commonly relied upon in the development of liposomes for controlled drug release, serving as an estimation of how they may perform in vivo as a drug delivery system [332].

### 5.3. Clinical Study Involving DDSs

Despite the fact that clinical application is the primary goal of this field and despite the development of a great deal of new knowledge, the number of DDSs that have reached the clinic is still relatively limited [333] (Table 1). During the translation process, researchers must overcome several obstacles, such as those connected to manufacture and storage, regulatory complexity, and cost. The challenges that do not apply to dry biodegradable polymers such as PLGA microspheres can arise due to the requirements of high water content in the polymeric MNs. Because some delivery systems are hydrated, terminal sterilization may be challenging. As a result, sterility must normally be confirmed for all source materials and production procedures [183]. A list of some clinical studies using different DDSs for the treatment of some obesity-related diseases is illustrated in Table S1 showing different clinical trials using DDs for the treatment of obesity and related diseases (ClinicalTrials.gov) (Supplementary File).

## 6. Conclusions

People are resistant to long-term medicine because they think that they do not require pharmacological treatment or because the adverse effects of pharmaceuticals are all well established. Developing anti-obesity drugs with fewer side effects could provide broader research and application prospects.

Drug delivery techniques are predicted to enhance obesity treatment. However, there is a pressing need to thoroughly comprehend the benefits and drawbacks of these formulations, as well as to get beyond the obstacles to clinical translation. In order to achieve greater scale-up and reproducibility in the industry, it is essential to understand the fate of the delivery system after delivery and the processes causing their toxicity. Pre-clinical testing is primarily used to select safe, effective formulations that possess the required biodistribution and pharmacokinetic properties, as well as to identify therapeutic potentials and dangers. It is critical to carefully analyse the biodistribution of drug carriers inside the body following administration and their interactions with target sites prior to utilization since the off-target toxicity of drug carriers could differ in comparison to the parent medicine. As a result, drug delivery methods must be researched further to decrease medicine dosage, minimize side effects, prevent patient fear of needles, prevent infection, and maintain regular fat loss while continually discovering new anti-obesity medications.

## Figures and Tables

**Table 5:** Techniques utilized for the assessment of MNs’ properties.

Characterization Technique	Description	Characteristics of MNs	Reference
Axial force	apply force to the needle’s tip in a vertical direction	determine the failure force of the needle tip	[242]
Transverse force	apply force into the needle base in parallel direction	determine the failure force of the needle base	[243]
Insertion test	apply the needles into rat, pig, or human skin	determine the actual force on skin and check the ability to release the drug	[85]

**Table 6:** Techniques utilized for the assessment of liposome properties.

Liposome Characteristics	Characterization Technique	References
Average particle	Dynamic light scattering (DLS) and microscope technology: scanning and transmission electron microscopy (SEM/TEM), cryogenic TEM (Cryo-TEM), and atomic force microscopy (AFM)	[288]
Zeta potential/surface charge	Electrophoretic mobility, DLS	[289]
Particle shape/morphology	TEM, Cryo-TEM, and AFM	[290]
Lamellarity	Cryo-TEM and 31P-NMR	[290]
Phase behaviour	X-ray diffraction (XRD), differential scanning calorimetry (DSC), and thermogravimetric analysis (TGA)	[291]
Encapsulation efficiency/drug release	Centrifugation, dialysis followed by drug content determination using chromatographic and/or spectrophotometric methods	[292]

**Table 7:** Different approaches used in delivery of drugs via MNs.

Delivery Approach	Description	Type of MNs	Reference
Poke and patch	Drug releases through micropores generated by MNs	Solid MNs	[303]
Coat and poke	Detachment of coating from the MN	Drug-coated MN	[304]
Poke and release	Drug diffuses and dissolves through the pores	Dissolving MN	[305]
Poke and flow	Drug flows out through the bore	Hollow MN	[306]

## Data Availability

Data will made available upon request.

## Supplementary Materials

The following supporting information can be downloaded at: https://www.mdpi.com/article/10.3390/pharmaceutics15112635/s1. Table S1. Different clinical trials using DDs for the treatment of obesity and related diseases (ClinicalTrials.gov). References [334,335,336,337,338,339,340,341,342,343,344,345,346,347,348,349,350,351,352,353,354,355,356,357,358,359,360,361,362,363,364,365,366,367,368,369] are cited in Supplementary Materials.

## Abbreviations

**Abbreviations**	**Proper Name**
DDS	Drug delivery system
WHO	World Health Organization
BMI	Body mass index
CNS	Central nervous system
MNs	Microneedles
NPs	Nanoparticles
FDA	US Food and Drug Administration
AT	Adipose tissue
WAT	White adipose tissue
BAT	Brown adipose tissue
UCP1	Uncoupling protein 1
ROSI	Rosiglitazone
GLP1	Glucagon-like peptide 1
PPAR	Peroxisome proliferator-activated receptor
EGCG	(-)-Epigallocatechin-3-gallate
GTE	Green tea extract
VAT	Visceral adipose tissue
PEG	Poly (ethylene glycol)
RAFT	Reversible addition–fragmentation transfer polymerization
ROP	Ring-opening polymerization
ROMP	Ring-opening metathesis polymerization
BBB	Blood–brain barrier
PK	Pharmacokinetics
PLGA	Poly(lactic-co-glycolic acid)
HFD	High-fat diet
LDL	Low-density lipoprotein
HDL	High-density lipoprotein
PYP	Polypyrrole
HA	Hyaluronic acid
DIO	Diet-induced obese
epiWAT	Epididymal white adipose tissue
igWAT	Inguinal white adipose tissue
DMNs	Dissolving microneedles
PCL	Polycaprolactone
MSH	Melanocyte-stimulating hormone
DBZ	Dibenzoazepine
AuNPs	Gold nanoparticles
NIR	Near-infrared
PA imaging	Photoacoustic imaging
RPE	Reverse-phase evaporation
TFH	Thin-film hydration
DLS	Dynamic light scattering
SEM	Scanning electron microscopy
TEM	Transmission electron microscopy
AFM	Atomic force microscopy
XRD	X-ray diffraction
DSC	Differential scanning calorimetry
TGA	Thermogravimetric analysis
SIF	Simulated intestinal fluid
PBS	Phosphate-buffered saline
MNA	Microneedle arrays
PVA	Polyvinyl alcohol
EPC	Egg yolk phosphatidylcholine
LITA	Liposome-encapsulated acetate
CHC	Carboxymethyl-hexanoyl chitosan
