# Supplementary material for: Anti-Obesity Drug Delivery Systems: Recent Progress and Challenges
Source: Pharmaceutics. 2023 Nov 16;15(11):2635. doi: 10.3390/pharmaceutics15112635 (PMC10674714; doi:10.3390/pharmaceutics15112635)
Supplement: Supplementary file 1 [file pharmaceutics-15-02635-s001.zip › pharmaceutics-2691019-supplementary.pdf]

Supplementary Information

Table S1. Different clinical trials using DDs for the treatment of obesity and related diseases (ClinicalTrials.gov)

Delivery system	Title	Status	Conditions	Interventions	Phases	References
hydrogels	Vestibular Stimulation to Trigger Adipose Loss (VeSTAL) Clinical Trial	completed	Obesity	Device: VeSTAL	not applicable	[1]
	Gelesis Glycemic Index Study	completed	Obesity, Diabetes, Pre-diabetes	Device: Gelesis200, Other: Water, only (placebo)	not applicable	[2]
liposomes	Liposomal Amphotericin B (AmBisome) Pharmacokinetics Given as a Single Intravenous Dose to Obese Patients (ASPEN)	completed	Morbid obesity	Drug: Administration of study drug	phase 4	[3]
	Transversus Abdominis Plane (TAP) Block Using Liposomal Bupivacaine in Metabolic and Bariatric Surgery Patients	not yet recruiting	Morbid Obesity, Bariatric Surgery Candidate	Drug: Liposomal bupivacaine, Drug: Bupivacaine Injection	phase 1	[4]

	Pain Control in Bariatric Patients: EXPAREL(R) vs. the On-Q(R) Pain Ball	completed	Obesity	Drug: EXPAREL, Drug: On-Q Pain Ball	phase 4	[5]
	Effects of Glutathione (an Antioxidant) and N-Acetylcysteine on Inflammation	completed	Obesity, Hyperlipidemia, Insulin Resistance, Hypertension	Dietary Supplement: Glutathione, Dietary Supplement: N-Acetylcysteine, Other: Placebo	not applicable	[6]
	The Effect of Curcumin on the Development of Prednisolone-induced Hepatic Insulin Resistance	terminated	Insulin Resistance, on-Alcoholic Fatty Liver Disease	Drug: Curcumin Liposome, Drug: Prednisolone, Drug: Placebos	not applicable	[7]
microparticles	Validation of Circulating Endothelial Cells and Microparticles in Youth	terminated	Childhood Obesity, Bariatric Surgery Candidate			[8]
	Embolization of Arterial Gastric Supply in Obesity	withdrawn	Morbid Obesity	Procedure: Emblization of the gastro-epiploic arcade	not applicable	[9]

	Bariatric Embolization Trial for the Obese Nonsurgical	not yet recruiting	Obesity, Weight Loss, Body Weight, Morbid Obesity	Procedure: Left gastric artery embolization	phase 1	[10]
	TIFACT Study - Tissue Factor Expression by Adipose Tissue in Extremely Obese Subjects.	completed	Obesity			[11]
	Metabolic Effects of Duodenal Jejunal Bypass Liner for Type 2 Diabetes Mellitus	completed	Type 2 Diabetes Mellitus, Obesity	Device: DJBL (Duodenal jejunal bypass liner, EndoBarrier)		[12]
	Beetroot Supplementation in Women Enjoying Exercise Together	recruiting	Aging, Postmenopause, Obesity	Dietary Supplement: Beetroot Juice, Other: Exercise Training	not applicable	[13]

	Homocysteine After Laparoscopic Roux-enY Gastric Bypass	completed	Obesity, Morbid, Homocystine; Metabolic Disorder, Bariatric Surgery Candidate, Folate Deficiency, Vitamin B 12 Deficiency	Procedure: laparoscopic long limb roux-en-Y gastric bypass		[14]
	Efficacy of Oral Vitamin D Supplementation in Adolescents and Young Adults.	unknown status	Overweight and Obesity, Overweight Adolescents, Vitamin D Deficiency	Dietary Supplement: 1000 IU D vitamin, Dietary Supplement: 200 IU D vitamin	not applicable	[15]
MNs	Insulin Delivery Using MNs in Type 1 Diabetes	completed	Type 1 Diabetes Mellitus	Device: Microneedle, Device: Subcutaneous insulin catheter	phase 2, phase 3	[16]
	Glucose Measurement Using Microneedle Patches	completed	Diabetes	Device: Microneedle patch, Device: Intravenous (IV) catheter, Device: Lancet	not applicable	[17]

	Enhanced Epidermal Antigen Specific Immunotherapy Trial -1	unknown status	Type 1 Diabetes	Drug: C19-A3 GNP	phase 1	[18]
	Pharmacokinetic Comparison of Intradermal Versus Sub-Cutaneous Insulin and Glucagon Delivery in Type 1 Diabetes	unknown status	Type 1 Diabetes	Procedure: Intradermal injection, Procedure: Subcutaneous injection	phase 2	[19]
	Multi-day (3) In-patient Evaluation of Intradermal Versus Subcutaneous Basal and Bolus Insulin Infusion	completed	Diabetes	Device: Subcutaneous delivery via Medtronic Quick-Set, Device: Intradermal delivery via the BD Research Catheter Set	phase 1, phase 2	[20]

	Pharmacokinetics/Dynamics of Basal (Continuous) Insulin Infusion Administered Either Intradermally or Subcutaneously	completed	DiabetesMellitus, Type 1diabètesMellitus, Type 2	Device: Intradermal insulin delivery: BD Research Catheter Set, Device: Subcutaneous insulin delivery:ACCU-CHEK Rapid-D Infusion Set	phase 2	[21]
	Suprachoroidal Injection of CLS-TA Alone or With Aflibercept in Subjects with Diabetic Macular Edema	completed	Diabetic Macular Edema	Drug : IVT Aflibercept, Drug : SC CLS-TA	phase 1, phase 2	[22]
NPs	Enhanced Epidermal Antigen Specific Immunotherapy Trial -1	unknown status	Type 1 Diabetes	Drug: C19-A3 GNP	phase 1	[18]
	Imaging Inflammation in Autoimmune Diabetes	completed	Diabetes Mellitus, Type 1			[23]

Capsaicin NP in Patient with Painful Diabetic Neuropathy	unknown status	Painful Diabetic Neuropathy	Drug: 0.75% capsaicin NP cream, Drug: Placebo	phase 2, phase 3	[24]
Host-microbiota-environment Interactions	not yet recruiting	Juvenile Idiopathic Arthritis, Diabetes type1|Inflammatory Bowel Diseases	Other: Stool sample		[25]
Clinical Application of Mesenchymal Stem Cells Seeded in Chitosan Scaffold for Diabetic Foot Ulcers	unknown status	Stem Cell Transplant	Drug: Stem Cell Product	phase 1	[26]
The Role of Eicosanoids in the Cardiovascular Actions of Inhaled NPs	active, not recruiting	Blood Biomarkers, Vasodilation, Blood Clotting, Lung Function, Healthy Volunteers	Other: diesel exhaust particulate, Other: carbon NPs, Other: small graphene oxide, Other: ultrasmall graphene oxide, Other: filtered air	not applicable	[27]

	the Accuracy and Safety of Coronary Artery Contrast-enhanced Magnetic Resonance Imaging with Polysaccharide Superparamagnetic Iron Oxide NP	recruiting	Coronary Heart Disease	Drug: domestic polysaccharide superparamagnetic iron oxide NP	phase 1	[28]
	Prevention of Restenosis Following Revascularization	terminated	Vascular Disease, Peripheral	Drug: NP Paclitaxel	phase 2	[29]
	Use of NP Paclitaxel (ABI-007) for the Prevention of In-Stent Restenosis	completed	Coronary Restenosis	Drug: NP Paclitaxel	phase 1, phase 2	[30]

Treatment of Patients with Atherosclerotic Disease with Methotrexate-associated to LDL Like NPs	recruiting	Atherosclerosis, Coronary Artery Disease, Inflammation	Drug: Methotrexate-LD, Drug: Placebo-LDE	phase 2, phase 3	[31]
Treatment of Patients with Atherosclerotic Disease with Paclitaxel-associated to LDL Like NPs	active, not recruiting	Coronary Artery Disease, Atherosclerosis, Inflammation	Drug: LDE-Paclitaxel, Drug: LDE-Placebo	phase 2, phase 3	[32]
Transforming NP Dressing for Management of Chronic Venous Ulcers	completed	Chronic Venous Hypertension with Ulcer	Combination Product: Transforming NP dressing, Combination Product: Conventional compression dressing	phase 4	[33]
Magnetic NPs System in Acute Coronary Syndrome	active, not recruiting	Acute Coronary Syndrome			[34]

Plasmonic Nanophotothermal Therapy of Atherosclerosis	completed	Stable Angin, |Heart Failure, Atherosclerosis, Multivessel Coronary Artery Disease	Procedure: Transplantation of NPs, Procedure: Transplantation of iron-bearing NPs, Device: Stenting	not applicable	[35]
Paclitaxel Albumin-Stabilized NP Formulation in Treating Patients with Relapsed or Refractory Multiple Myeloma	terminated	Refractory Plasma Cell Myeloma	Drug: Paclitaxel Albumin-Stabilized NP Formulation, Other: Laboratory Biomarker Analysis	phase 2	[36]

References

[1] Gelesis, I. inVentiv Health Clinical, Gelesis Glycemic Index Study. 2017. Available online: https://ClinicalTrials.gov/show/NCT03124134 (accessed on).

[2] Neurovalens Ltd., S.D. University of California, University of Ulster, Exploristics, Compliance Solutions Ltd., Vestibular Stimulation to Trigger Adipose Loss (VeSTAL) Clinical Trial. 2019. Available online: https://ClinicalTrials.gov/show/NCT03640286 (accessed on).

[3] Liposomal Amphotericin B (AmBisome) Pharmacokinetics Given as a Single Intravenous Dose to Obese Patients (ASPEN), (n.d.). Available online: https://ClinicalTrials.gov/show/NCT02320604 (accessed on).

[4] Transversus Abdominis Plane (TAP) Block Using Liposomal Bupivacaine in Metabolic and Bariatric Surgery Patients, (n.d.). Available online: https://ClinicalTrials.gov/show/NCT05537883 (accessed on).

[5] Pain Control in Bariatric Patients: EXPAREL(R) vs. the On-Q(R) Pain Ball, (n.d.). Available online: https://ClinicalTrials.gov/show/NCT02142829 (accessed on).

[6] Effects of Glutathione (an Antioxidant) and N-Acetylcysteine on Inflammation, (n.d.). Available online: https://ClinicalTrials.gov/show/NCT01550432 (accessed on).

[7] The Effect of Curcumin on the Development of Prednisolone-induced Hepatic Insulin Resistance, (n.d.). Available online: https://ClinicalTrials.gov/show/NCT04315350 (accessed on).
[8] Validation of Circulating Endothelial Cells and Microparticles in Youth, (n.d.). Available online: https://ClinicalTrials.gov/show/NCT01508598 (accessed on).
[9] Embolization of Arterial Gastric Supply in Obesity, (n.d.). Available online: https://ClinicalTrials.gov/show/NCT04207424 (accessed on).
[10] Bariatric Embolization Trial for the Obese Nonsurgical, (n.d.). Available online: https://ClinicalTrials.gov/show/NCT03601273 (accessed on).
[11] TIFACT Study—Tissue Factor Expression by Adipose Tissue in Extremely Obese Subjects, (n.d.). Available online: https://ClinicalTrials.gov/show/NCT00379704 (accessed on).
[12] Metabolic Effects of Duodenal Jejunal Bypass Liner for Type 2 Diabetes Mellitus, (n.d.). Available online: https://ClinicalTrials.gov/show/NCT02800668 (accessed on).
[13] Beetroot Supplementation in Women Enjoying Exercise Together, (n.d.). Available online: https://ClinicalTrials.gov/show/NCT04370756 (accessed on).
[14] Homocysteine After Laparoscopic Roux-enY Gastric Bypass, (n.d.). Available online: https://ClinicalTrials.gov/show/NCT03489538 (accessed on).
[15] Efficacy of Oral Vitamin D Supplementation in Adolescents and Young Adults, (n.d.). Available online: https://ClinicalTrials.gov/show/NCT04377386 (accessed on).
[16] Emory University, Thrasher Research Fund, Insulin Delivery Using Microneedles in Type 1 Diabetes. 2008. Available online: https://ClinicalTrials.gov/show/NCT00837512 (accessed on).
[17] Emory University, Glucose Measurement Using Microneedle Patches. 2017. Available online: https://ClinicalTrials.gov/show/NCT02682056 (accessed on).
[18] Cardiff University, Enhanced Epidermal Antigen Specific Immunotherapy Trial-1. 2016. Available online: https://ClinicalTrials.gov/show/NCT02837094 (accessed on).
[19] Massachusetts General Hospital, Pharmacokinetic Comparison of Intradermal Versus Sub-cutaneous Insulin and Glucagon Delivery in Type 1 Diabetes. 2012. Available online: https://ClinicalTrials.gov/show/NCT01684956 (accessed on).
[20] Becton, D. Company, Multi-day (3) In-Patient Evaluation of Intradermal Versus Subcutaneous Basal and Bolus Insulin Infusion. 2012. Available online: https://ClinicalTrials.gov/show/NCT01557907 (accessed on).
[21] Becton, D. Company, Pharmacokinetics/Dynamics of Basal (Continuous) Insulin Infusion Administered Either Intradermally or Subcutaneously. 2010. Available online: https://ClinicalTrials.gov/show/NCT01061216 (accessed on).

[22] Biomedical, I.C. Suprachoroidal Injection of CLS-TA Alone or with Aflibercept in Subjects with Diabetic Macular Edema. 2016. Available online: https://ClinicalTrials.gov/show/NCT02949024 (accessed on).
[23] Imaging Inflammation in Autoimmune Diabetes, (n.d.). Available online: https://ClinicalTrials.gov/show/NCT00585936 (accessed on).
[24] Capsaicin Nanoparticle in Patient With Painful Diabetic Neuropathy, (n.d.). Available online: https://ClinicalTrials.gov/show/NCT01125215 (accessed on).
[25] Host-Microbiota-Environment Interactions, (n.d.). Available online: https://ClinicalTrials.gov/show/NCT05176795 (accessed on).
[26] Clinical Application of Mesenchymal Stem Cells Seeded in Chitosan Scaffold for Diabetic Foot Ulcers, (n.d.). Available online: https://ClinicalTrials.gov/show/NCT03259217 (accessed on).
[27] The Role of Eicosanoids in the Cardiovascular Actions of Inhaled Nanoparticles, (n.d.). Available online: https://ClinicalTrials.gov/show/NCT03659864 (accessed on).
[28] The Accuracy and Safety of Coronary Artery Contrast-enhanced Magnetic Resonance Imaging With Polysaccharide Superparamagnetic Iron Oxide Nanoparticle, (n.d.). Available online: https://ClinicalTrials.gov/show/NCT05032937 (accessed on).
[29] Prevention of Restenosis Following Revascularization, (n.d.). Available online: https://ClinicalTrials.gov/show/NCT00518284 (accessed on).
[30] Use of Nanoparticle Paclitaxel (ABI-007) for the Prevention of In-Stent Restenosis, (n.d.). Available online: https://ClinicalTrials.gov/show/NCT00124943 (accessed on).
[31] Treatment of Patients With Atherosclerotic Disease With Methotrexate-associated to LDL Like Nanoparticles, (n.d.). Available online: https://ClinicalTrials.gov/show/NCT04616872 (accessed on).
[32] Treatment of Patients With Atherosclerotic Disease With Paclitaxel-associated to LDL Like Nanoparticles, (n.d.). Available online: https://ClinicalTrials.gov/show/NCT04148833 (accessed on).
[33] Transforming Nanoparticle Dressing For Management of Chronic Venous Ulcers, (n.d.). Available online: https://ClinicalTrials.gov/show/NCT04793074 (accessed on).
[34] Magnetic Nanoparticles System in Acute Coronary Syndrome, (n.d.). Available online: https://ClinicalTrials.gov/show/NCT02226523 (accessed on).
[35] Plasmonic Nanophotothermal Therapy of Atherosclerosis, (n.d.). Available online: https://ClinicalTrials.gov/show/NCT01270139 (accessed on).
[36] Paclitaxel Albumin-Stabilized Nanoparticle Formulation in Treating Patients With Relapsed or Refractory Multiple Myeloma, (n.d.). (accessed on)
